# Effectiveness of a scalable group-based education and monitoring program, delivered by health workers, to improve control of hypertension in rural India: A cluster randomised controlled trial

**DOI:** 10.1371/journal.pmed.1002997

**Published:** 2020-01-02

**Authors:** Dilan Giguruwa Gamage, Michaela A. Riddell, Rohina Joshi, Kavumpurathu R. Thankappan, Clara K. Chow, Brian Oldenburg, Roger G. Evans, Ajay S. Mahal, Kartik Kalyanram, Kamakshi Kartik, Oduru Suresh, Nihal Thomas, Gomathyamma K. Mini, Pallab K. Maulik, Velandai K. Srikanth, Simin Arabshahi, Ravi P. Varma, Rama K. Guggilla, Fabrizio D’Esposito, Thirunavukkarasu Sathish, Mohammed Alim, Amanda G. Thrift

**Affiliations:** 1 Department of Medicine, School of Clinical Sciences at Monash Health, Monash University, Melbourne, Victoria, Australia; 2 George Institute for Global Health, University of New South Wales, Sydney, New South Wales, Australia; 3 University of Sydney, Sydney, New South Wales, Australia; 4 George Institute for Global Health, New Delhi, India; 5 Achutha Menon Centre for Health Science Studies, Sree Chitra Tirunal Institute for Medical Sciences and Technology, Trivandrum, Kerala, India; 6 Department of Cardiology, Westmead Hospital, Sydney, New South Wales, Australia; 7 Melbourne School of Population and Global Health, University of Melbourne, Melbourne, Victoria, Australia; 8 Cardiovascular Disease Program, Biomedicine Discovery Institute and Department of Physiology, Monash University, Melbourne, Victoria, Australia; 9 School of Public Health and Preventative Medicine, Monash University, Melbourne, Victoria, Australia; 10 Nossal Institute for Global Health, Melbourne School of Population and Global Health, University of Melbourne, Melbourne, Victoria, Australia; 11 Rishi Valley Rural Health Centre, Chittoor District, Andhra Pradesh, India; 12 Department of Endocrinology, Diabetes & Metabolism, Christian Medical College, Vellore, Tamil Nadu, India; 13 Global Institute of Public Health, Ananthapuri Hospitals and Research Institute, Trivandrum, Kerala, India; 14 George Institute for Global Health, Oxford University, Oxford, United Kingdom; 15 Peninsula Clinical School, Central Clinical School, Monash University, Frankston, Victoria, Australia; 16 Department of Population Medicine and Civilization Diseases Prevention, Faculty of Medicine, Division of Dentistry and Division of Medical Education in English, Medical University of Bialystok, Bialystok, Poland; 17 Centre for Population Health Sciences, Lee Kong Chian School of Medicine, Nanyang Technological University, Singapore; 18 University of Central Lancashire, Preston, United Kingdom; Johns Hopkins University Bloomberg School of Public Health, UNITED STATES

## Abstract

**Background:**

New methods are required to manage hypertension in resource-poor settings. We hypothesised that a community health worker (CHW)–led group-based education and monitoring intervention would improve control of blood pressure (BP).

**Methods and findings:**

We conducted a baseline community-based survey followed by a cluster randomised controlled trial of people with hypertension in 3 rural regions of South India, each at differing stages of epidemiological transition. Participants with hypertension, defined as BP ≥ 140/90 mm Hg or taking antihypertensive medication, were advised to visit a doctor. In each region, villages were randomly assigned to intervention or usual care (UC) in a 1:2 ratio. In intervention clusters, trained CHWs delivered a group-based intervention to people with hypertension. The program, conducted fortnightly for 3 months, included monitoring of BP, education about hypertension, and support for healthy lifestyle change. Outcomes were assessed approximately 2 months after completion of the intervention. The primary outcome was control of BP (BP < 140/90 mm Hg), analysed using mixed effects regression, clustered by village within region and adjusted for baseline control of hypertension (using intention-to-treat principles). Of 2,382 potentially eligible people, 637 from 5 intervention clusters and 1,097 from 10 UC clusters were recruited between November 2015 and April 2016, with follow-up occurring in 459 in the intervention group and 1,012 in UC. Mean age was 56.9 years (SD 13.7). Baseline BP was similar between groups. Control of BP improved from baseline to follow-up more in the intervention group (from 227 [49.5%] to 320 [69.7%] individuals) than in the UC group (from 528 [52.2%] to 624 [61.7%] individuals) (odds ratio [OR] 1.6, 95% CI 1.2–2.1; *P =* 0.001). In secondary outcome analyses, there was a greater decline in systolic BP in the intervention than UC group (−5.0 mm Hg, 95% CI −7.1 to −3.0; *P <* 0.001) and a greater decline in diastolic BP (−2.1 mm Hg, 95% CI −3.6 to −0.6; *P <* 0.006), but no detectable difference in the use of BP-lowering medications between groups (OR 1.2, 95% CI 0.8–1.9; *P =* 0.34). Similar results were found when using imputation analyses that included those lost to follow-up. Limitations include a relatively short follow-up period and use of outcome assessors who were not blinded to the group allocation.

**Conclusions:**

While the durability of the effect is uncertain, this trial provides evidence that a low-cost program using CHWs to deliver an education and monitoring intervention is effective in controlling BP and is potentially scalable in resource-poor settings globally.

**Trial registration:**

The trial was registered with the Clinical Trials Registry-India (CTRI/2016/02/006678).

## Abstract 2

Amanda Thrift and colleagues reveal the benefits of education around hypertension and improved lifestyle guidance for patients in rural India with high blood pressure.

## Author summary

**Why was the study done?:**

- Many regions of the world have inadequate or inaccessible health resources and health professionals to diagnose, monitor, or manage hypertension.
- This gap could potentially be addressed by using non-physician community health workers (CHWs), who reside in the community and are available in sufficient numbers.

**What did the researchers do and find?:**

- In a cluster randomised controlled trial in 3 very diverse regions in rural India, 637 participants with hypertension from 5 clusters were recruited and randomised to the intervention, and 1,097 with hypertension from 10 clusters were recruited and randomised to usual care.
- Every 2 weeks, CHWs educated people in the intervention clusters about hypertension and measured their blood pressure.
- Blood pressure declined an average of 5.0/2.1 mm Hg more in the intervention group than the usual care group, and control of blood pressure improved.

**What do these findings mean?:**

- Our approach overcomes limitations in access to healthcare by utilising a CHW workforce that not only is local, but requires very little training.
- This low-cost group-based education and monitoring intervention is a potentially scalable approach that could be implemented across other diverse settings in rural India, and globally.
- Use of this approach may help reduce the emergence of cardiovascular diseases in low-resource settings.

## Introduction

Hypertension is the largest contributor to the global burden of disease, and has a prevalence that has almost doubled over the last 25 years, from 442 million in 1990 to 874 million in 2015 [1]. This growth has occurred most rapidly in low- and middle-income countries (LMICs) [1], which now account for the majority of those with hypertension (66%) [2]. India, with rapidly rising prevalence of hypertension also occurring in rural regions [3], is now well on its way to becoming the global “hypertension capital” [4].

Controlling hypertension is paramount for reducing risks of adverse outcomes, but there are significant barriers to its control, particularly in rural regions [5]. For example, awareness of having hypertension is significantly less among those with hypertension in rural (mean 25%) than urban (mean 42%) India [5], and differs markedly across rural regions [5]. In addition, patients often do not receive evidence-based care for hypertension, with a recent review providing evidence that only 25% of people with hypertension in rural India were receiving treatment, and only 11% had their blood pressure (BP) controlled [5]. Such inadequate care is partly due to a shortage of doctors (below 1:20,000 in some regions) [6] and doctor absenteeism in public health centres [7]. Other barriers include the cost of treatment and the lack of availability of medications [8,9].

Sharing tasks between community health workers (CHWs) and doctors is a promising and potentially scalable approach for delivering care for hypertension in hard-to-reach settings [10], as it improves access to healthcare and reduces costs associated with treatment [11]. Only 7 studies have incorporated this approach to managing hypertension in LMICs [12–18], and in another study, it was unclear whether the staff sharing the tasks were nurses or CHWs [19]. Furthermore, only 4 of the studies included a randomised comparator [12,13,17,18]. While there were reductions in BP and improved control of hypertension, the interventions cited in these studies were all resource-intensive individualised approaches. None incorporated a group-based approach, which has been shown to reduce costs in other diseases [20], thereby enhancing scalability.

We aimed to determine whether a CHW-led group-based education and monitoring program for the management of hypertension is effective, both individually and by region, in rural India. We used a cluster design to facilitate our group-based approach.

## Methods

### Study design

The study was conducted in 2 stages. Initially, a baseline community-based survey was conducted to identify people with hypertension. This was followed by an open-label cluster randomised controlled trial (cRCT) of an intervention program to manage hypertension, as outlined in our published study protocol [21].

### Study setting

The study was conducted in 3 rural regions of South India with differing economic and epidemiological profiles. The Trivandrum region in Kerala has a relatively high life expectancy of 76.4 years [22] and more than 90% literacy [23]. In the Rishi Valley region, located in Chittoor District near the southwestern border of Andhra Pradesh, the population largely comprises subsistence farmers. Approximately half the population in this region has no formal schooling [24]. The Western Godavari study region is economically intermediate between these 2 other regions [23]. In 2011, approximately 75% of the population in West Godavari were literate [23].

Ethics approval was obtained from Sree Chitra Tirunal Institute for Medical Sciences and Technology (Trivandrum, India; SCT/IEC-484/July-2013), the Centre for Chronic Disease Control (CCDC-IEC-09-2012), Christian Medical College (Vellore, India), the Health Ministry Screening Committee of the Government of India (58/4/1F/CHR/2013/NCD II), and Monash University (CF13/2516–2013001327).

### Participants

**Fig 1:**
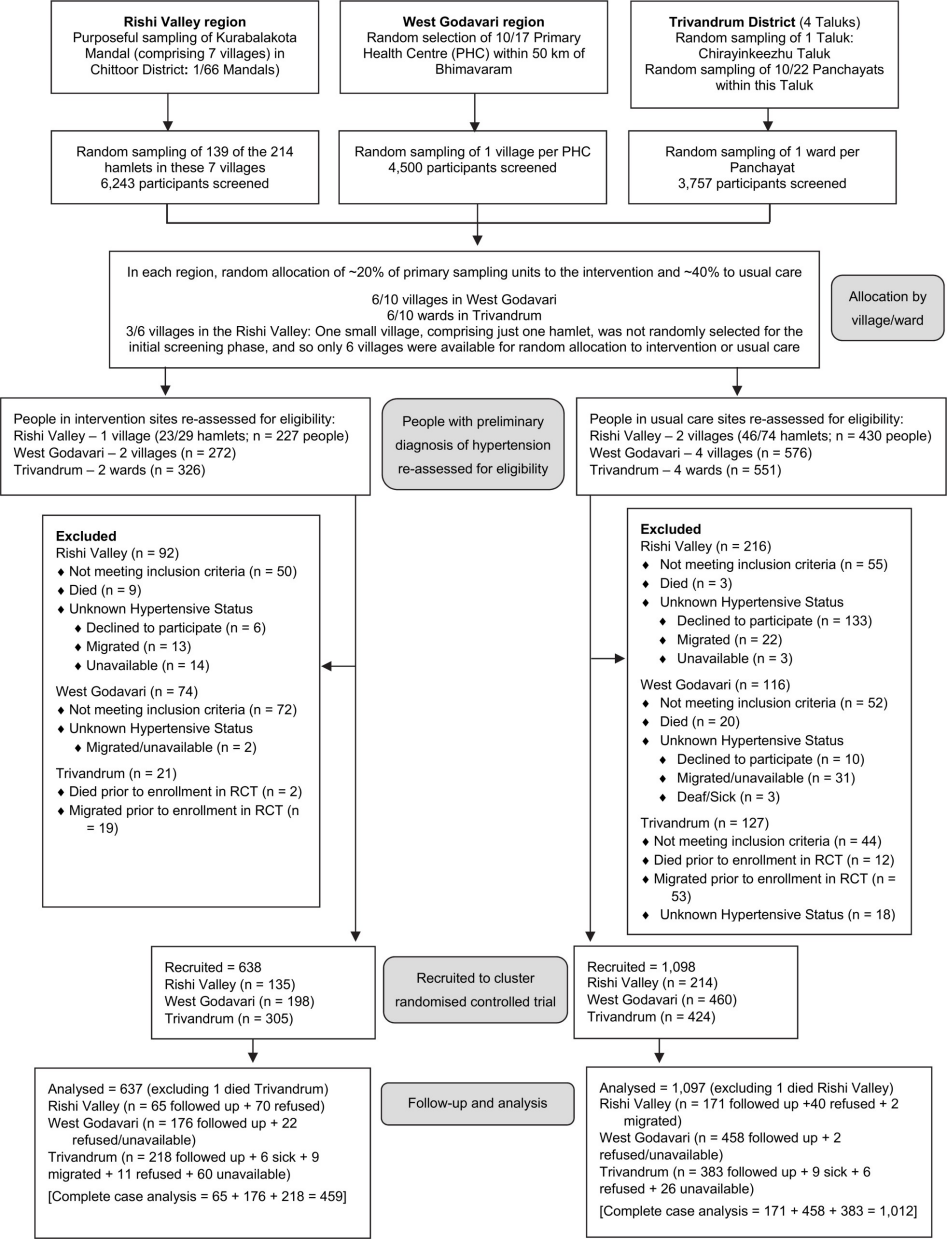
Flow diagram of trial participants. ‘Not meeting inclusion criteria’ refers to those not categorised as hypertensive when re-assessed for eligibility. BP, blood pressure; PHC, primary health centre; RCT, randomised controlled trial.

In each region, primary sampling units (PSUs) were wards, villages, or hamlets, the last being clusters of approximately 10 to 200 houses geographically separated within a larger village (collectively referred to as “villages” hereafter) (Fig 1). Members of the investigator team of each region randomly selected the PSUs. To randomly identify potential participants, polling lists were used in Trivandrum, whereas a list of residents compiled following a census we conducted was used in West Godavari (see [21] for more details).

Age and sex stratification was used to obtain approximately equal numbers of males and females in the age categories 18–24, 25–34, 35–44, 45–54, 55–64, and 65+ years in each PSU. In Trivandrum, 10 wards were surveyed, with approximately 375 participants in each (3,757 total). In West Godavari, 10 villages were surveyed, with approximately 450 participants in each (4,500 total). In the Rishi Valley, 139 hamlets, located within 7 villages, were randomly selected (6,243 participants). Thus, 14,500 participants were surveyed from a total of 27 clusters in 3 regions of India. Individual informed consent was obtained, via a signature or thumb print, following full explanation of the study to potential participants prior to enrolment in the baseline survey and again prior to randomisation into the cRCT.

### Participant baseline assessments

Prior to commencing the study, we engaged with local village leaders in each region. Then, a team of trained research assistants conducted a baseline community-based survey between September 2013 and December 2015. We measured BP and anthropometry and conducted interviews to elicit details on use of medications, demographics, lifestyle behaviours, and access to healthcare. Arterial BP was measured after the participant had been seated quietly for at least 15 minutes. Three measurements were taken at 3-minute intervals using the appropriate cuff size and a calibrated digital automatic BP monitor (OMRON HEM-907, OMRON Healthcare, Kyoto, Japan) according to the WHO STEPS protocol, modified only by using the right arm for all measurements [25]. Measurement continued until 2 consecutive readings differed by <10 mm Hg systolic and <6 mm Hg diastolic, with a maximum of 5 measurements. The mean of the last 2 consecutive measurements was considered the baseline BP level for each participant.

We measured height to the nearest 0.1 cm using a portable stadiometer (213, Seca, Hamburg, Germany) and weight to the nearest 0.1 kg using a portable digital weighing scale (9000SV3R, Salter, Kent, UK). Waist and hip circumference were measured using a spring-loaded tension tape (Gulick M-22C, Patterson Medical, Illinois, US) in a private setting. In accordance with the WHO STEPS protocol [25], waist circumference was measured at the midpoint between the lowest rib and upper point of the iliac crest and at the end of normal expiration, while hip circumference was measured at the maximum protrusion of the buttocks.

At the time of the baseline survey, all participants with systolic BP (SBP) ≥ 140 mm Hg and/or diastolic BP (DBP) ≥ 90 mm Hg were informed they may have hypertension and were advised to visit a doctor to have their BP re-checked.

### Randomisation and eligibility

Participants re-assessed for eligibility to be recruited to the cRCT were those identified as potentially hypertensive in the baseline community-based survey. Because of limited resources, we did not recruit or follow-up participants in all PSUs. Instead the lead investigator (AGT), located off site, used a computer-generated random sequence to randomly allocate 20% of PSUs to the intervention and 40% to usual care (UC) in each region (Fig 1). Eligibility of all potential participants within these PSUs was determined prior to enrolment [21], using the following definition for hypertension: (1) self-report of an existing diagnosis of hypertension; (2) use of antihypertensive medications; (3) SBP ≥ 140 mm Hg and/or DBP ≥ 90 mm Hg in the baseline community-based survey, with a subsequent diagnosis of hypertension by their primary healthcare provider; or (4) SBP ≥ 140 mm Hg and/or DBP ≥ 90 mm Hg both in the baseline survey and at another measurement prior to recruitment to the cRCT. All people with verified hypertension, i.e., satisfying any of the above criteria, were invited to participate by the study field staff, and comprised the intention-to-treat group.

### CHWs and training

The intervention program was conducted by accredited social health activists (ASHAs), who are CHWs who reside in most villages in rural India. We employed 14 ASHAs who were already working in this role in the region [26]. In 1 village that had no ASHA, we specifically employed someone for this role.

Prior to commencement of the intervention, CHWs were trained to deliver the community-based program over a 5-day course [21,26]. They were remunerated for their time in accordance with government salary scales (for specific details of this remuneration please refer to [21]). This included time for training and for delivering the intervention. No further training was provided during the 3-month intervention period. However, study supervisors, who were specifically employed for the study, reviewed the content and activities with CHWs after each intervention session.

### Patient and public involvement

Participants and the public were involved in the shaping of the intervention program in a number of ways. First, the results of focus group discussion and preliminary analyses of the baseline survey drove content for the educational materials. These interactions highlighted poor knowledge of hypertension and poor access to health services. CHWs who were involved in the pilot training and local clinicians in the Rishi Valley (K. Kar. and K. Kal.) also provided feedback on this content, enabling refinement of the educational resources. In addition, in October 2014 a stakeholder meeting was held in which experts from the Ministry of Health and the Indian Council of Medical Research and an independent researcher provided input as to how this approach could fit within the Indian health system.

### Intervention

The intervention consisted of 6 fortnightly sessions of ~90 minutes, held within the villages (clusters) in which the participants resided, and delivered over 3 months. Each participant in an intervention cluster was allocated to 1 of 32 groups in which the intervention was delivered: 6 in the Rishi Valley, 14 in West Godavari, and 12 in Trivandrum. CHWs encouraged group members to attend all 6 sessions. At the start of each fortnightly session, CHWs measured BP and weighed all participants to assist with self-management. During each session, they delivered education about hypertension and how to manage it, in the local language [21,26]. This included details about adhering to medications and the importance of making lifestyle changes, such as increasing physical activity and following a healthier diet. Pictorial flipcharts were used as education aids, and handouts were provided to participants to use at home. Full details of the education provided, including all session flipcharts and handouts, are available online (doi: 10.4225/03/5967f9a94970d [English version]). Those whose BP was controlled were provided the same education as those whose BP was not controlled, with positive feedback provided when BP levels were maintained below 140/90 mm Hg.

### Usual care

The UC group was not informed of the intervention program. They were contacted once between the baseline survey and the final follow-up to determine their eligibility, i.e., their hypertensive status was confirmed, as for the intervention group.

### Blinding

As this was a behavioural intervention, neither the workers delivering the program nor the participants in the intervention group could be blinded to the intervention. Participants in the UC group were unaware of the intervention. The outcome assessors were the same trained research assistants who conducted the baseline survey. They were also not blinded to treatment group, as they monitored the CHWs during delivery of the intervention.

### Outcomes

Participant outcomes were collected at a follow-up visit occurring approximately 2 months after the final session of the group-based education and monitoring program. The primary outcome was a change from baseline in the proportion of people with controlled hypertension (BP < 140/90 mm Hg), as outlined in our clinical trial registration. Secondary BP outcomes, as listed in our published protocol [21], included change in SBP and DBP from baseline, measured in an identical way to the baseline survey in accordance with the WHO STEPS protocol [25]. Other outcomes comprised changes in use of antihypertensive medications, body mass index (BMI), waist–hip ratio (WHR), and lifestyle behaviours (physical activity, fruit and salt intake, tobacco smoking, and alcohol consumption).

### Sample size

Our original sample size was based on a mean SBP among people with hypertension of 147 mm Hg (SD 22) and a 6–mm Hg reduction with the intervention, slightly less than that seen in an earlier trial of hypertension in a developed country [27]. We further estimated 100 participants per cluster (30 clusters) and presumed an intraclass correlation coefficient (ICC) of 0.047, giving a design effect of 5.653 [28]. At a significance level of 5% and a power of 80%, and adjusting for clustering (i.e., adjusting for the design effect), we estimated requiring 1,097 people with hypertension per study arm. However, as we had insufficient resources to include all of the clusters in the subsequently designed cRCT, our eventual sample size was projected to be less than this, leading us to initially propose this as a feasibility study. Towards the end of the baseline survey, a preliminary analysis of mean baseline SBP in 15 clusters revealed that the ICC among those with SBP ≥ 140 mm Hg was 0.01, with 37% having controlled hypertension. Estimating an average cluster size of 120 yielded a design effect of 2.19 (1 + [119 × 0.01] = 2.19). Using a 1:2 ratio for the intervention versus UC and a power of 80% to detect a difference in the proportion with controlled BP of 37% in UC versus 50% in the intervention group resulted in an initial required sample size of 170 in the intervention group and 339 in UC. Multiplying by the design effect (i.e., 2.19), to ensure adequate power for a cRCT, resulted in a requirement of 372 in the intervention group and 742 in UC. From the baseline survey, we estimated that we would have approximately 600 in the intervention group and 1,200 in UC. This sample size would give us 94% power to detect the same level of effect (i.e., difference in the proportion with controlled BP of 37% in UC versus 50% in the intervention group), hence providing the rationale to analyse this as a stand-alone cRCT. Sample size was determined for the whole sample and not for subgroups, i.e., site or sex.

### Data management and analysis

Data from questionnaires were captured into an electronic database using TeleForm version 10.5 (Cardiff Software, CA, US). The data were cleaned by identifying outliers. Potential errors in these outliers were checked with the original forms and by seeking clarification from the research sites. Errors identified in this way were then corrected.

In the analysis we followed intention-to-treat principles (Stata IC/11.2, StataCorp, College Station, TX, US). ANOVA was used to determine whether baseline characteristics (continuous variables) differed significantly between treatment groups, and between regions, and whether the differences between the treatment groups varied according to region. Tukey’s test was applied to determine which regions differed. Student’s unpaired *t* test was used to detect whether differences existed between the treatment groups in each of the 3 regions, so a Bonferroni correction was applied to protect against increased risk of type I error. Differences in categorical variables between regions and treatment groups were analysed using chi-squared tests with Bonferroni correction to account for multiple comparisons between and within regions. Two-tailed *P* ≤ 0.05 was considered statistically significant.

The primary outcome comprised mixed effects logistic regression analyses, clustered by village within region and adjusted for baseline control of BP, conducted to determine the odds for control of BP for the intervention versus UC group. We used similar analyses to determine the odds of prescription of antihypertensive medication (a secondary analysis). Separate analyses were conducted for women and men.

In sensitivity analyses that included all eligible participants, the outcome data from individuals who dropped out of the study were imputed using multiple imputation by chained equations as some variables were binary and others continuous. Twenty imputed datasets were created for each relevant outcome variable. Details of the proportion of missing observations imputed for each variable and the variables used in each imputation model are provided in the footnotes to tables and figures.

For imputed outcomes, differences in categorical variables between groups were initially assessed using a 2-sample test of proportions, while linear regression was used to determine differences in continuous variables. Further, sensitivity analyses using the same methods were conducted in those whose BP was uncontrolled at baseline, but without baseline adjustment.

Mixed effects linear regression analyses, adjusted for baseline control of BP and clustered by village within region, were used to determine the effectiveness of the intervention in reducing SBP and DBP (secondary analyses). Separate analyses were conducted for women and men.

The trial was registered with the Clinical Trials Registry–India (CTRI; CTRI/2016/02/006678). We applied for registration on 28 September 2015, approximately 2 months before the first patient was enrolled. As delays in having a trial approved are very common in India, it is usual practice to commence recruiting patients before the final registration number is received. We obtained official approval from the CTRI on 25 February 2016 (CTRI/2016/006678), notably with no changes to the protocol from our original application.

## Results

In total, 2,382 residents were identified as potentially eligible to participate in this cRCT. Since the baseline survey, 46 had died and 140 had migrated, leaving 2,196 potentially eligible (Fig 1). Among these, 273 did not meet the inclusion criteria for verified diagnosis of hypertension, while in 187 the eligibility status could not be ascertained (149 refused to participate and 38 either were unavailable or their status was unknown), resulting in 1,736 recruited. In the villages randomly allocated to the intervention, the 3-month group-based education and monitoring program commenced first in West Godavari (November 2015) and last in Trivandrum (April 2016). Final follow-up of 1,471 participants occurred between January and September 2016, with 200 (11.5%) refusing or unavailable for the final outcome assessment. In sensitivity analyses, outcome variables were imputed for these participants. Two participants died, and so were not included in the outcome analyses.

**Fig 2:**
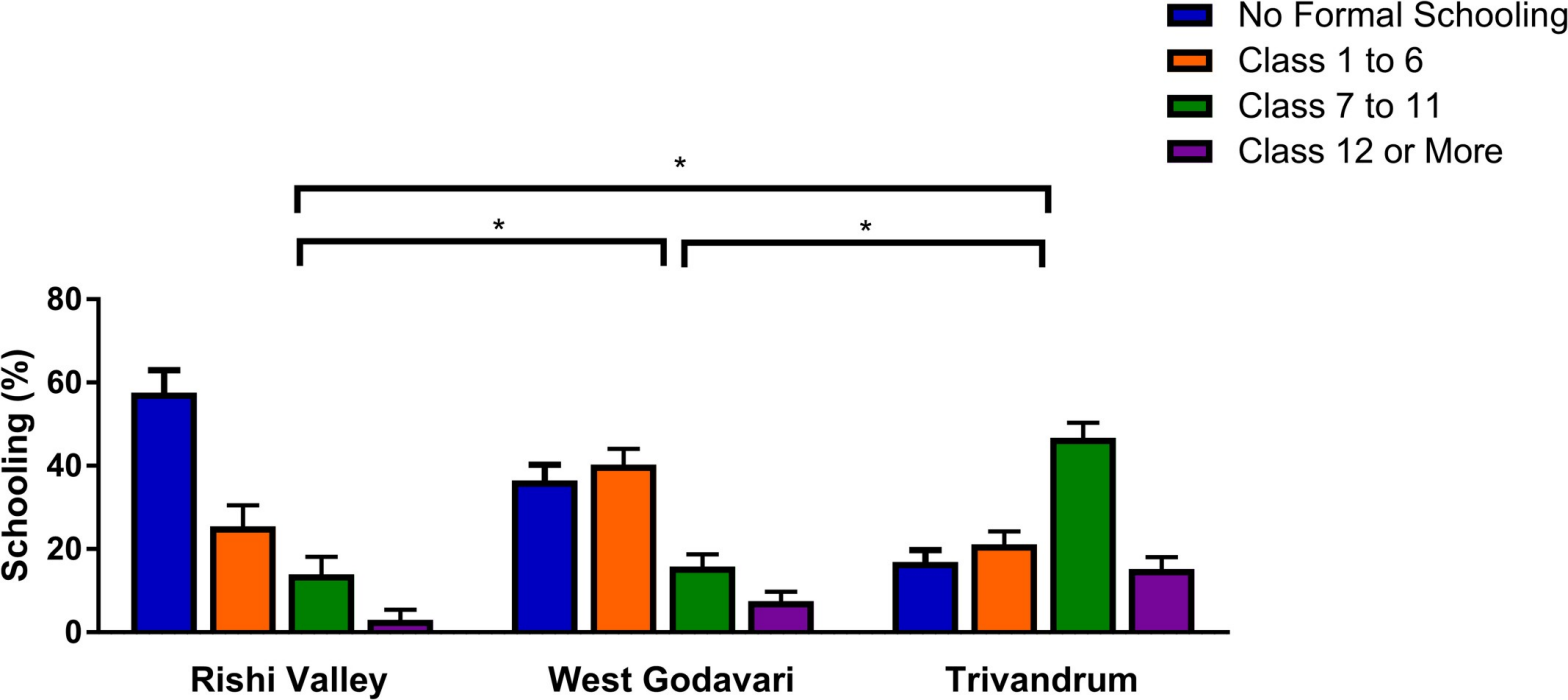
Educational attainment of participants by region. *n* = 1,711 (18 missing observations for the Rishi Valley and 5 missing for West Godavari, with none imputed). *P < 0.01, with Bonferroni correction for specific contrasts between each of the 3 regions.

Participants from the Rishi Valley region had poorer educational attainment, literacy, and other markers of socioeconomic position than the other 2 regions (Fig 2; S1 Table). Educational attainment was greatest in Trivandrum, with 62.0% of participants completing at least class 7 (Fig 2). This compares with 23.3% in West Godavari and 17.0% in the Rishi Valley (both *P* for difference from Trivandrum < 0.001; Fig 2). Participants in the Rishi Valley also self-reported greater difficulty in accessing healthcare than the other 2 sites (*P* < 0.001).

**Fig 3:**
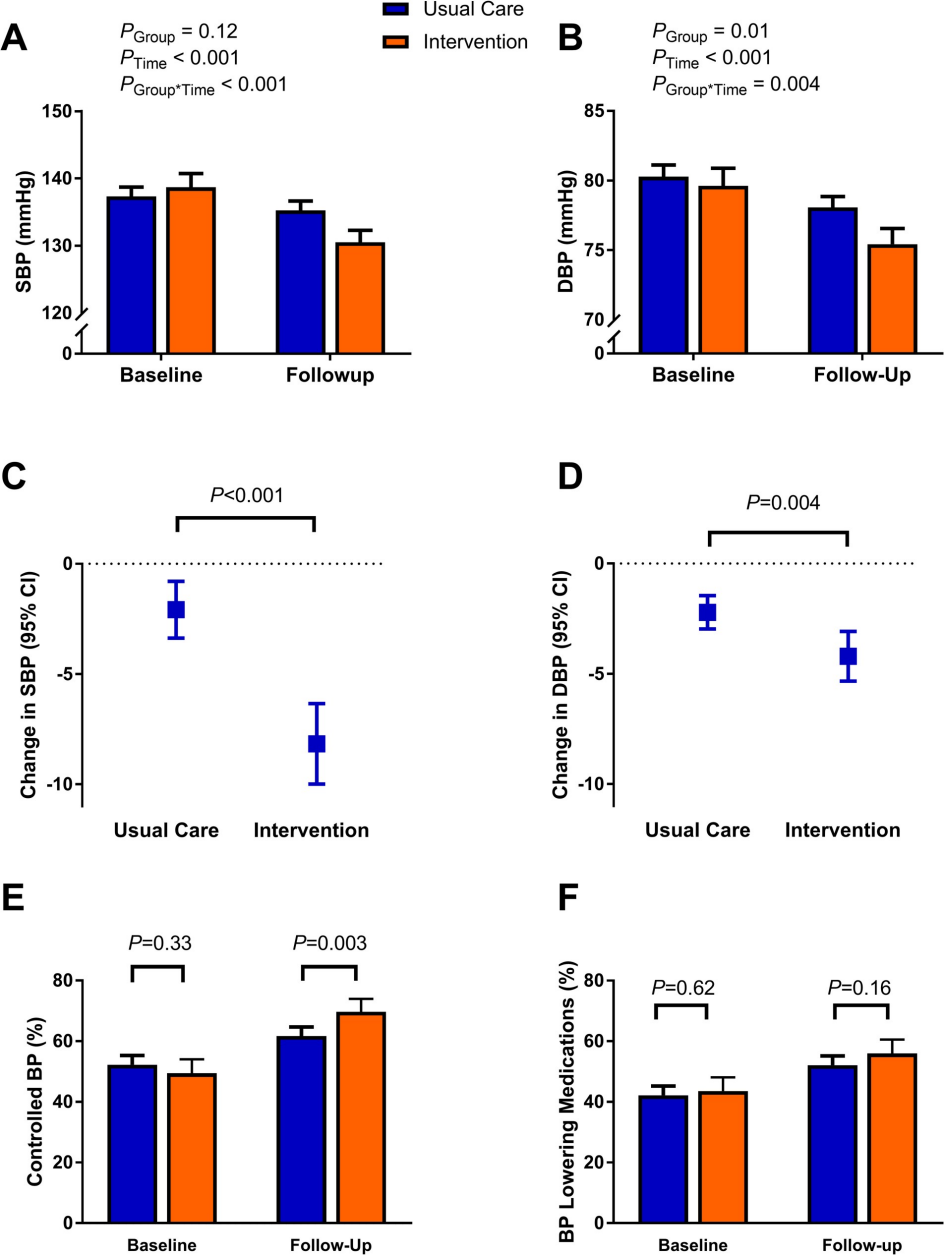
Summary of study findings. SBP (A) and DBP (B) at baseline and follow-up according to study group. Change in SBP (C) and DBP (D) in the usual care and intervention groups. Proportion of patients with controlled BP (E) and taking BP-lowering medications (F) at baseline and follow-up according to study group. *P* values for categorical variables were generated using a test for 2-sample differences in proportions (E and F), while differences in continuous variables were generated using linear regression (A–D). Error bars show 95% confidence limits. BP, blood pressure; DBP, diastolic blood pressure; SBP, systolic blood pressure.

The mean age of participants was 56.8 years, and 58.2% were female (Table 1). Overall, baseline characteristics of participants were balanced between the intervention and UC groups, except that participants in the intervention group reported eating more servings of fruit per week. Importantly, control of BP, and mean SBP and DBP, were similar between groups overall (Table 1; Fig 3) and within regions (S2 Table) but differed between regions (S1 Fig; S2 Table). Baseline mean SBP was approximately 9 mm Hg greater in participants in the Rishi Valley than in the other 2 regions (*P <* 0.001), while baseline mean DBP differed between all 3 regions (*P <* 0.001), being greatest in the Rishi Valley (85.1 mm Hg), least in Trivandrum (78.5 mm Hg), and intermediate in West Godavari (80.3 mm Hg). There were also large differences between regions in BMI and WHR, which were least in the Rishi Valley and greatest in Trivandrum (*P* for difference < 0.001; S2 Table).

**Table 1:** Baseline characteristics of the intention-to-treat sample.

Variable	Intervention *n* = 637	UC *n* = 1,097
Age (years), mean (SD)	56.6 (14.3)	56.9 (13.7)
Female, *n* (%)	373 (58.7)*	633 (57.9)^§^
SBP (mm Hg), mean (SD)	140.5 (22.7)	137.8 (22.2)
DBP (mm Hg), mean (SD)	80.4 (13.7)	80.6 (13.9)
Controlled hypertension, *n* (%)	277 (43.5)	549 (50.1)
Antihypertensive medication, *n* (%)	242 (38.0)	445 (40.6)
Body mass index (kg/m^2^), mean (SD)	24.5 (4.8)^†^	24.8 (5.0)^¶^
Waist hip ratio, mean (SD)	0.92 (0.09)^¶^	0.92 (0.09)^§^
Physical activity per day (METS), mean (SD)	928 (947)^§^	860 (896)^§^
Fruit (weekly servings), mean (SD)	3.3 (5.8)^§^	2.5 (3.3)^‖^
Vegetables (weekly servings), mean (SD)	11.3 (7.9)	10.5 (6.4)
Teaspoons of salt added/day, mean (SD)	0.05 (0.09)^¶^	0.06 (0.13)
Adding extra salt to food, *n* (%)	227 (36.0)^¶^	410 (38.6)
Current smoking, *n* (%)	96 (15.3)^¶^	165 (15.1)^¶^
Current alcohol use, *n* (%)	68 (10.8)^¶^	145 (13.3)^¶^

None of these baseline data were imputed. Control of hypertension is defined as SBP < 140 mm Hg and DBP < 90 mm Hg; control may be achieved with use of antihypertensive medications or changing lifestyle.

*One missing observation.

^†^Two missing observations.

^‡^Three missing observations.

^§^Four missing observations.

^‖^Five missing observations.

^¶^Six to eight missing observations. Salt added to food: 34 missing observations in UC. The data for servings of vegetables in Trivandrum had some errors that could not be resolved. Therefore, there are no data for weekly servings of vegetables in this region. These data on servings of vegetables rely on 329 observations in the intervention group and 668 in the UC group.

DBP, diastolic blood pressure; METS, metabolic equivalent tasks; SBP, systolic blood pressure; SD, standard deviation; UC, usual care.

### Primary outcome

In the primary outcome analysis, which was clustered by village and study region, there was 1.6-fold (95% CI 1.2 to 2.1; *P =* 0.001) better control of hypertension in the intervention group than in the UC group at follow-up (Table 2; Fig 3E). Similar results were obtained in sensitivity analyses that included those who were recruited but not followed up (S3 Table). In further sensitivity analyses limited to those whose BP was uncontrolled at baseline, similar results were obtained for the overall group, but findings appeared to be limited to men (S4 Table). Control of hypertension improved more in the intervention than UC group in 2 of the 3 regions (S5 Table).

**Table 2:** Effects of intervention on control of hypertension (primary outcome) and prescription of antihypertensive medication (secondary outcome) in people with hypertension: complete case analysis.

Outcome	Number of participants		Change from baseline to follow-up*		*P* value	Adjusted odds ratio (95% CI)^†^	*P* value
	Intervention	UC^‡^	Intervention	UC			
**Overall**							
Control of hypertension	459	1,011	93 (20.3)	96 (9.5)	<0.001	1.6 (1.2–2.1)	0.001
Prescribed antihypertensive medication	459	1,012	57 (12.4)	100 (9.9)	0.14	1.2 (0.8–1.9)	0.34
**Women**							
Control of hypertension	280	596	52 (18.6)	49 (8.2)	<0.001	1.6 (1.1–2.2)	0.01
Prescribed antihypertensive medication	280	597	38 (13.6)	45 (7.5)	0.004	1.5 (0.9–2.2)	0.09
**Men**							
Control of hypertension	178	411	41 (23.0)	48 (11.7)	<0.001	1.6 (1.1–2.5)	0.02
Prescribed antihypertensive medication	178	411	19 (10.7)	54 (13.1)	0.40	0.9 (0.5–1.7)	0.85

There are 4 missing observations for sex in the UC group and 1 in the intervention group.

*Change in control of hypertension was obtained by subtracting the number of people with control of hypertension at baseline from the number with control at follow-up. The same approach was applied to prescription of antihypertensive medication. Positive number demonstrates improvement.

^†^Odds ratios obtained using mixed effects logistic regression, clustered by village and study region. For control of hypertension, the dependent variable was control of hypertension at follow-up, with adjustment for control of hypertension at baseline (ICC: overall, 0.002; women, 0.003; men, 0.008). The same approach was applied to prescription of antihypertensive medication (ICC: overall, 0.04; women, 0.02; men, 0.09).

^‡^A person who did not have blood pressure measured at follow-up had details of medications, and so there is an extra observation for the latter analysis.

ICC, intraclass correlation coefficient; UC, usual care.

### Secondary outcomes

In secondary outcomes, there was no evidence for greater uptake in the use of BP-lowering medications with the intervention (*P =* 0.31; Fig 3F; Table 2), except in West Godavari (*P <* 0.01; S5 Table). Interestingly, there was greater uptake of BP-lowering medications in women in the intervention than UC group, although not in adjusted analyses (Table 2). This improvement in women appeared to be driven by the improvement observed in West Godavari (S6 Table). There was no evidence for a change in use of medications by men in any region, although sample sizes were small.

Overall, SBP declined by 8.2 mm Hg in the intervention group, 6.1 mm Hg more than in the UC group (Table 3; Fig 3A and 3C). This finding was similar in imputation analyses that included those who were not followed up (S7 Table). The decline in SBP was greatest in the Rishi Valley, being 13.6 mm Hg in the intervention group, (S8 Table). This change in SBP was greater than in the other 2 regions (*P <* 0.001; distribution shown in S2 Fig). Also, in contrast to the other 2 regions, participants in the Rishi Valley region showed a large decline in SBP in the UC group.

**Table 3:** Effects of intervention on secondary outcomes in people with hypertension: complete case analysis.

Outcome	Number of participants		Unadjusted mean change (95% CI)		Unadjusted net mean change (95% CI)	*P* value	Adjusted net mean change (95% CI)	*P* value
	Intervention	UC	Intervention *n* = 459	UC *n* = 1,012				
**Overall**								
SBP (mm Hg)	459	1,011	−8.2 (−10.0 to −6.3)	−2.1 (−3.4 to −0.8)	−6.1 (−8.4 to −3.8)	<0.001	−5.0 (−7.1 to −3.0)*	<0.001
DBP (mm Hg)	459	1,011	−4.2 (−5.3 to −3.1)	−2.2 (−3.0 to −1.4)	−2.0 (−3.4 to 0.6)	0.004	−2.1 (−3.6 to −0.6)^†^	<0.006
**Women**								
SBP (mm Hg)	280	596	−6.7 (−8.8 to −4.6)	−1.5 (−3.1 to 0.2)	−5.2 (−8.0 to −2.4)	<0.001	−4.8 (−7.2 to −2.3)^‡^	<0.001
DBP (mm Hg)	280	596	−2.6 (−3.9 to −1.3)	−1.7 (−2.7 to 0.8)	−0.9 (−2.5 to 0.7)	0.29	−1.3 (−2.7 to 0.0)^§^	0.06
**Men**								
SBP (mm Hg)	178	411	−10.5 (−13.8 to −7.2)	−3.1 (−5.2 to −1.0)	−7.4 (−11.3 to −3.5)	<0.001	−6.3 (−10.3 to −2.2)^‖^	0.002
DBP (mm Hg)	178	411	−6.8 (−8.8 to −4.8)	−3.0 (−4.3 to −1.7)	−3.8 (−6.2 to −1.4)	0.002	−3.9 (−7.0 to −0.8)^¶^	0.014

For change values, negative number demonstrates improvement. Adjusted analyses were conducted using mixed effects linear regression, clustered by region and village.

*Adjusted for age, sex, SBP at baseline, use of antihypertensive medications, change in body mass index, and alcohol use; 37 missing observations due to missing variables. ICC = 0.022.

^†^Adjusted for age, sex, DBP at baseline, education, use of antihypertensive medications, change in body mass index, fruit per week, and alcohol use; 49 missing observations due to missing variables. ICC = 0.020.

^‡^Adjusted for age, SBP at baseline, regular visits to doctor, use of antihypertensive medications, change in body mass index, and adding salt to food; 32 missing observations due to missing variables. ICC < 0.001.

^§^Adjusted for age, DBP at baseline, education, regular visits to doctor, use of antihypertensive medications, and change in body mass index; 13 missing observations due to missing variables. ICC = 0.010.

^‖^Adjusted for age, SBP at baseline, education, use of antihypertensive medications, and alcohol use; 15 missing observations due to missing variables. ICC = 0.021.

^¶^Adjusted for age, DBP at baseline, education, regular visits to doctor, use of antihypertensive medications, and alcohol use; 15 missing observations due to missing variables. ICC = 0.045.

DBP, diastolic blood pressure; ICC, intraclass correlation coefficient; SBP, systolic blood pressure; UC, usual care.

Similar to the pattern observed for SBP, DBP also declined more in the intervention group (4.2 mm Hg) than the UC group (2.2 mm Hg; Fig 3B and 3D; Table 3). Also, in contrast to the other 2 regions, there was a large decline in DBP in the UC group in the Rishi Valley region (S8 Table; S1 Fig; distribution shown in S3 Fig).

There was no evidence for an effect of the intervention on BMI, WHR, physical activity, or fruit consumption (Table 4), but the intervention appeared to result in greater reductions in extra salt added to food (*P =* 0.003), smoking (*P <* 0.001), and alcohol consumption (*P* < 0.001) than in the UC group (Table 5).

**Table 4:** Changes in risk factors from baseline to follow-up in the intervention and UC groups (continuous variables).

Variable	Mean change (95% confidence interval)		*P* value
	Intervention *n* = 637	UC *n* = 1,097	
BMI (kg/m^2^)^1^	0.17 (0.03 to 0.31)*	0.23 (0.12 to 0.34)^§^	0.50
WHR^1^	0.006 (0.001 to 0.012)^‖^	0.003 (−0.001 to 0.006)^§^	0.29
Physical activity (METS) per day^2^	104 (89 to 119)^†^	108 (98 to 118)^†^	0.65
Fruit (weekly servings)^2^	0.03 (−0.47 to 0.53)^†^	0.48 (0.24 to 0.72)^‡^	0.08

Change in BMI between baseline and follow-up was imputed for 260 observations (using BMI at baseline); change in WHR was imputed for 257 observations (using WHR and BMI at baseline); change in physical activity per day was imputed for 259 observations (using physical activity at baseline); change in fruit consumption per week was imputed for 259 observations (using fruit consumption at baseline).

^1^Negative number demonstrates improvement.

^2^Positive number demonstrates improvement.

*Two missing observations.

^†^Four missing observations.

^‡^Five missing observations.

^§^Six missing observations.

^‖^Seven missing observations.

BMI, body mass index; METS, metabolic equivalent tasks; UC, usual care; WHR, waist–hip ratio.

**Table 5:** Changes in risk factors from baseline to follow-up in the intervention and UC groups (categorical variables).

Variable	Change in number of individuals (%)		*P* value
	Intervention	UC	
**Overall**	***n* = 637**	***n* = 1,097**	
Change in adding extra salt to food	−69 (−11.0)^†^	−73 (−6.9)^‖^	0.003
Change in current smoking	−18 (−2.9)^†^	−6 (−0.6)^†^	<0.001
Change alcohol use in last 30 days	−7 (−1.1)^†^	3 (0.2)^†^	<0.001
**Women**	***n* = 373**	***n* = 633**	
Change in adding extra salt to food	−37 (10.1)^†^	−45 (7.3)^§^	0.13
Change in current smoking	−6 (−1.7)^†^	1 (0.2)^†^	0.001
Change alcohol use in last 30 days	−1 (−0.1)^†^	3 (0.5)*	0.19
**Men**	***n* = 263**	***n* = 460**	
Change in adding extra salt to food	−32 (−12.3)*	−29 (−6.5)^‡^	0.009
Change in current smoking	−12 (−4.7)*	−7 (−1.6)*	0.014
Change alcohol use in last 30 days	−6 (−2.4)*	−1 (−0.1)*	0.006

Negative number demonstrates improvement. Data for salt use at follow-up were imputed for 254 observations (using salt use at baseline); data for smoking at follow-up were imputed for 258 observations (using smoking at baseline and sex); data for alcohol consumption at follow-up were imputed for 258 observations (using alcohol consumption at baseline and sex). Change in adding salt to food was obtained by subtracting the number of people reporting adding salt to food at baseline from the number adding salt to food at follow-up. This same approach was applied for the other variables in the table.

*Two to four missing observations.

^†^Five to eight missing observations.

^‡^Sixteen missing observations.

^§^Eighteen missing observations.

^‖^Thirty-four missing observations.

UC, usual care.

There appeared to be some sex differences in the effects of the intervention on BP. While SBP was reduced in both women and men (Table 3 and S9 Table), the reduction in DBP appeared to occur in men only.

**Fig 4:**
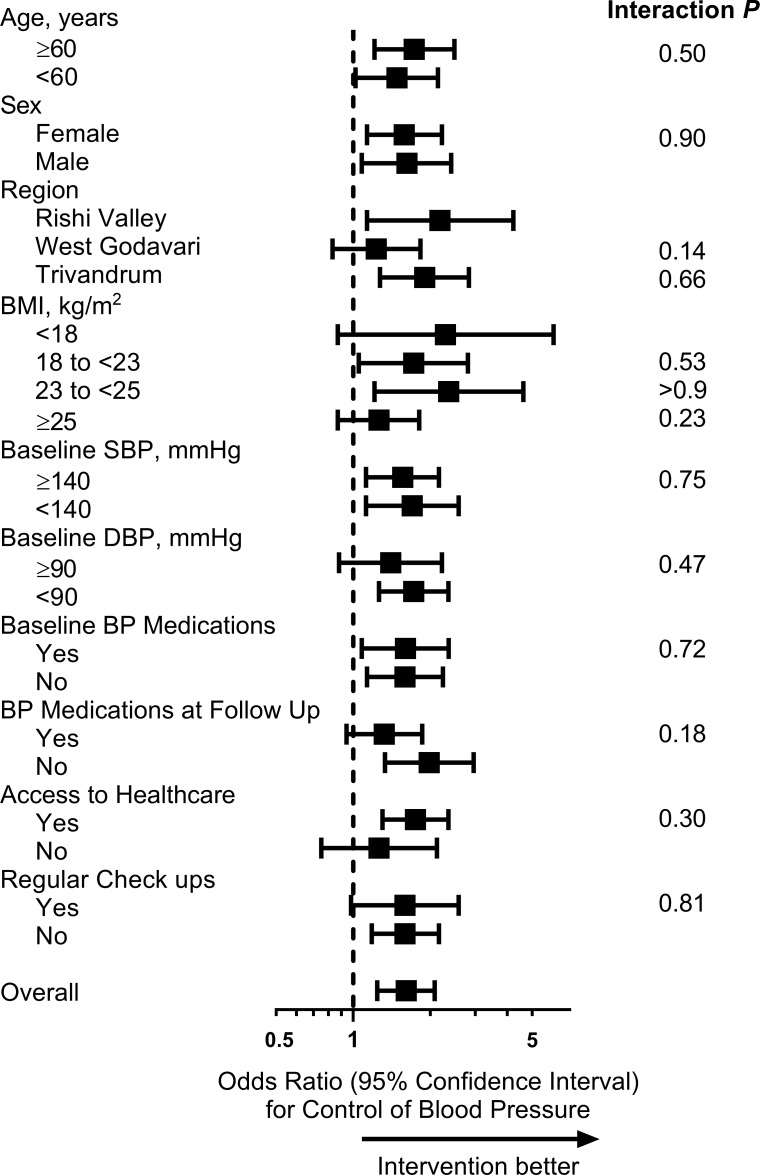
Forest plot of differences in control of BP by group, according to different characteristics of the sample (complete case analysis). The dashed line represents the line of no effect. Symbols show point estimates, and error bars show 95% confidence limits. *P* values indicate subgroup interactions (obtained using logistic regression). The following variables had missing data: BMI, 7; access to healthcare, 9; and regular check-ups, 7. BMI, body mass index; BP, blood pressure; DBP, diastolic blood pressure; SBP, systolic blood pressure.

There was no evidence that the effectiveness of the intervention on control of BP differed according to age or other differences in characteristics (Fig 4). This finding appeared to be similar for women and men separately (S4 Fig).

## Discussion

This cRCT, conducted in rural India, provides evidence that a group-based education and monitoring program, delivered by health workers, is effective in reducing SBP and DBP and in improving control of hypertension. The intervention was effective in all 3 regions, which are at differing stages of the economic and epidemiological transition, indicating that the intervention could be effective and scalable across rural India. With an estimated prevalence of hypertension of 25% among adults in rural India [5], our intervention is thus potentially scalable to more than 100 million adults with hypertension living in rural India.

Our intervention has enormous potential for reducing BP and improving control of hypertension in poor and hard-to-reach settings. This was just a 3-month intervention, yet the BP reduction attained was comparable to that observed in clinical trials of BP-lowering medications, equating to a 20% reduction in the risk of coronary heart disease and vascular death [29]. Apart from the education provided to intervention participants in a group setting, at each session participants also had their BP measured. This ongoing monitoring may have empowered participants to determine whether and how their lifestyle changes resulted in tangible benefits [30]. This approach also provides motivation for continuing behavioural changes or adopting new ones [30], and this may be one of the mechanisms for the success of the intervention. Furthermore, the program included several components that were evidence-based for improving the control of hypertension. These components included improving the adherence to antihypertensive medications [31], providing regular monitoring of BP [30], and encouraging lifestyle changes such as losing excess weight and increasing physical activity [32].

Trained professionals are not always available in rural and remote settings [6,7]. Thus, training the existing workforce in specific tasks, such as monitoring BP and providing education and support, that do not require clinical decision-making but can be implemented using evidence-based protocols, enables a reorganisation of health tasks to improve access and minimise costs [10]. Although other investigators have also shown the effectiveness of task-shifting management of hypertension to CHWs [12,15–17], major limitations included that these studies involved intensive, time-consuming individual-based interventions [12,13,15–17], lacked a UC group [14–16], or provided home BP monitors (equipment unaffordable to the poor) [12]. Our approach, using a group-based intervention, provides a novel, effective, and potentially cheaper and sustainable alternative to managing hypertension in disadvantaged and hard-to-reach settings.

Our findings confirm and extend those from other task-sharing intervention programs of the effect of education programs in LMICs. He et al. reported a 6.6/5.4–mm Hg greater reduction in BP in participants exposed to a CHW-led multicomponent education and coaching program, home BP monitoring, text messaging, and better trained physicians than in those receiving UC [12]. A similar decrease of 5 mm Hg of SBP was reported by Jafar et al. in Pakistan in a group exposed to home health education and upskilled general practitioners, compared to other groups [17], and Neupane et al. reported a 4.9–mm Hg greater decrease in SBP in a group in Nepal with home-monitoring and education, compared to UC [13,17]. However, all 3 studies were of individualised-care approaches, which are likely to be more resource-intensive than our group-based approach.

Simple surveillance may be an incentive to patients to take steps to reduce their BP levels in regions with poor access to healthcare. There was a large improvement in SBP and DBP in the UC group in the Rishi Valley region, a decline of 9.5/6.8 mm Hg. Following the baseline community-based survey, participants who had BP levels of ≥140/90 mm Hg were informed that their BP was high and that this might put them at risk of other diseases, and were advised to visit a doctor. While some of the observed decline in BP could be regression to the mean, it might also suggest that BP surveillance itself can help control BP in regions with poor access to healthcare.

It was notable that the effect of the intervention did not appear to be attributable to new prescription of antihypertensive medication at follow-up. Potentially, BP may have declined with the adoption of healthy lifestyle habits, with some evidence of reductions in alcohol consumption, an effect largely seen in men as few women consumed alcohol at baseline. There was some evidence that adding salt to food declined, but as most of the salt in the diet is added in the cooking process, it is unlikely that reducing salt at the table would explain the BP reductions observed.

### Strengths and limitations of the study

There were some limitations to our study that may influence the interpretation of our findings. Although the sample size was large overall, the number of participants in the Rishi Valley was less than half the number of participants in either of the other 2 regions. Of the individuals with hypertension identified in the baseline community-based survey in each region, we recruited approximately 20% to the intervention group and 40% to the UC group. However, the baseline prevalence of hypertension was the least in the Rishi Valley, and even though we surveyed a larger number of people, it was not sufficient to ensure similar group sizes. In addition, a total of 263 individuals dropped out of the study, most of whom were in the intervention group. This raises potential issues around the intention-to-treat approach, with subsequent potential biases, as discussed by Giraudeau and Ravaud [33]. We minimised bias by randomising clusters after the baseline community-based survey was conducted, and so we had baseline data on all those who participated in the baseline survey. Although some people then dropped out, we included these individuals in a sensitivity analysis by imputing their outcome, thereby reducing the potential bias of excluding these individuals who dropped out. Of note, the findings for the 2 regions with a larger number of individuals who dropped out are consistent with those from the West Godavari region, where only 24 individuals dropped out, adding to the credence of our findings. There were also no empty clusters. We also acknowledge that both the 3-month intervention and the follow-up period of 6–8 weeks, between the last session of the program and assessment of outcomes, were relatively short. Because of this, we cannot determine whether there is a long-lasting improvement in controlling hypertension from our group-based education and monitoring program. It is also possible that there was some selection bias as the control of BP was relatively high at baseline, being approximately 50%. Indeed, in those who did not participate, only 19.9% had their BP controlled at baseline, indicating that extra efforts should be made to improve the reach of the intervention. There could potentially have been some post-randomisation selection bias as individuals were recruited following randomisation of their village. However, potential participants were blinded to the recruitment status of their village at the time that their eligibility was established and their recruitment sought, thereby reducing this potential bias. Another limitation of our study is that it was not possible to blind the outcome assessors to the treatment allocation of the individuals as the assessors were instrumental in observing the fidelity of the intervention. However, we reduced the impact of detection bias on our main outcome measures by using digital automated BP monitors, thereby limiting the influence of the perceptions of the outcome assessors that might have arisen with the use of auscultation. A further limitation is that, for logistic reasons, details about salt intake were asked of individuals only about salt added at the table. However, as most of the salt consumed is added during cooking, these details would have been better obtained from the person responsible for the cooking. Thus, the data we generated on salt consumption should be interpreted with caution. Some of the reduction in BP observed may be attributable to the Hawthorne effect, whereby participants alter behaviours just because they are being observed. However, this does not discount the difference that we observed between the UC and intervention groups, comprising a randomised comparison.

There are several strengths to our study, including the cluster-randomised allocation of the PSUs to facilitate delivery of the group-based intervention, and the consistency in the delivery of the program in all 3 regions. The PSUs were geographically separated and had different CHWs, thereby minimising contamination between groups. Another major strength is our inclusion of 3 very diverse regions of rural India using the available workforce, so that our findings may be generalisable to a large portion of rural India. Additionally, the sample size of our project was large, thereby reducing the likelihood of a chance finding. Furthermore, our study included a UC comparator, which enabled us to minimise the effects of other variables. Only 4 prior trials of CHW-led task-shifting intervention programs to treat hypertension included a clearly defined UC group [12,13,17,18], so our study is one of the few to have a robust comparator to test the effectiveness of the intervention.

### Conclusion

Our program was effective in increasing the proportion who had control of hypertension, and in reducing both SBP and DBP. Simple surveillance also appeared to result in reduced BP in these rural settings. Our findings add to the limited research on task-shifting interventions for treating hypertension in LMICs. In particular, we have shown that this intervention is applicable across very diverse rural settings, so there is considerable potential to implement and scale up across rural India and potentially other resource-poor regions in other countries. Future programs incorporating comprehensive collection of data on changes in medication use, changes to lifestyle behaviours, and knowledge of hypertension may help tease out which factors are best targeted to improve the control of hypertension in these settings.

## Supporting information

S1 CONSORT Checklist CONSORT checklist for cluster randomised trials. (DOCX)

S1 Fig Complete case analysis of BP outcomes between the intervention and UC groups, by site and overall. (A) Percent with controlled BP, (B) percent with BP-lowering medications, (C) mean SBP, (D) mean DBP, and (E) change in BP (mean change in mm Hg, and 95% confidence intervals). *P* values for categorical variables were generated using chi-squared tests (A and B) or linear regression for continuous variables (C–E), with Bonferroni correction for specific contrasts between each of the 3 regions. Error bars show 95% confidence limits.
(TIF)

S2 Fig Distribution of change in SBP between baseline and follow-up, by intervention group and site (complete case analysis). (TIF)

S3 Fig Distribution of change in DBP between baseline and follow-up, by intervention group and site (complete case analysis). (TIF)

S4 Fig Forest plot of between-group differences in control of BP in women and men, according to different characteristics of the sample (complete case analysis). (A) Women; (B) men. The dashed line represents the line of no effect. Symbols show point estimates, and error bars show 95% confidence limits. *P* values indicate subgroup interactions (obtained using logistic regression).
(TIF)

S1 Table Socioeconomic characteristics of participants in the intervention and UC groups in the 3 regions. (DOCX)

S2 Table Baseline characteristics of participants with hypertension in the intervention and UC groups. (DOCX)

S3 Table Effects of the intervention on control of hypertension (primary outcome) and prescription of antihypertensive medication (secondary outcome) in people with hypertension: Imputation analysis using intention-to-treat principles. (DOCX)

S4 Table Sensitivity analysis of the effects of the intervention on the primary outcome (control of hypertension) in people with hypertension, excluding those with controlled hypertension at baseline. (DOCX)

S5 Table Changes in risk factors from baseline to follow-up in the intervention and UC groups (categorical variables). (DOCX)

S6 Table Changes in control of BP and use of antihypertensive medications from baseline to follow-up in women and men in the intervention and UC groups. (DOCX)

S7 Table Effects of the intervention on secondary outcomes in people with hypertension: Imputation analysis using intention-to-treat principles. (DOCX)

S8 Table Changes in risk factors from baseline to follow-up in the intervention and UC groups (continuous variables). (DOCX)

S9 Table Changes in BP from baseline to follow-up in women and men in the intervention and UC groups. (DOCX)

## Abbreviations

BMI: body mass index
BP: blood pressure
CHW: community health worker
cRCT: cluster randomised controlled trial
DBP: diastolic blood pressure
LMICs: low- and middle-income countries
PSU: primary sampling unit
SBP: systolic blood pressure
UC: usual care
WHR: waist–hip ratio

## References

[1] Forouzanfar MH, Liu P, Roth GA, Ng M, Biryukov S, Marczak L, et al Global burden of hypertension and systolic blood pressure of at least 110 to 115 mm Hg, 1990–2015. JAMA. 2017;317(2):165–82. 10.1001/jama.2016.19043 28097354

[2] NCD Risk Factor Collaboration. Worldwide trends in blood pressure from 1975 to 2015: a pooled analysis of 1479 population-based measurement studies with 19.1 million participants. Lancet. 2017;389(10064):37–55. 10.1016/S0140-6736(16)31919-5 27863813 PMC5220163

[3] Gupta R. Convergence in urban-rural prevalence of hypertension in India. J Hum Hypertens. 2016;30(2):79–82. 10.1038/jhh.2015.48 26108364

[4] Joshi SR, Parikh RM. India—diabetes capital of the world: now heading towards hypertension. J Assoc Physicians India. 2007;55:323–4. 17844690

[5] Anchala R, Kannuri NK, Pant H, Khan H, Franco OH, Di Angelantonio E, et al Hypertension in India: a systematic review and meta-analysis of prevalence, awareness, and control of hypertension. J Hypertens. 2014;32(6):1170–7. 10.1097/HJH.0000000000000146 24621804 PMC4011565

[6] Central Bureau of Health Intelligence. National health profile 2015. New Delhi: Central Bureau of Health Intelligence; 2015 [cited 2018 Nov 9]. Available from: https://www.thehinducentre.com/multimedia/archive/02557/National_Health_Pr_2557764a.pdf.

[7] Muralidharan K, Chaudhury N, Hammer J, Kremer M, Rogers FH. Is there a doctor in the house? Medical worker absence in India. San Diego: University of California, San Diego, Department of Economics; 2011 [cited 2018 Nov 9]. Available from: http://econweb.ucsd.edu/~kamurali/papers/Working%20Papers/Is%20There%20a%20Doctor%20in%20the%20House%20-%2012%20April,%202011.pdf.

[8] Busingye D, Arabshahi S, Evans RG, Srikanth VK, Kartik K, Kalyanram K, et al Factors associated with awareness, treatment and control of hypertension in a disadvantaged rural Indian population. J Hum Hypertens. 2017;31(5):347–53. 10.1038/jhh.2016.85 28054571

[9] Attaei MW, Khatib R, McKee M, Lear S, Dagenais G, Igumbor EU, et al Availability and affordability of blood pressure-lowering medicines and the effect on blood pressure control in high-income, middle-income, and low-income countries: an analysis of the PURE study data. Lancet Public Health. 2017;2(9):e411–9. 10.1016/S2468-2667(17)30141-X 29253412

[10] World Health Organization. Taking stock: task shifting to tackle health worker shortages. Geneva: World Health Organization; 2007 [cited 2018 Nov 9]. Available from: http://www.who.int/healthsystems/task_shifting/TTR_tackle.pdf?ua=1.

[11] Joshi R, Alim M, Kengne AP, Jan S, Maulik PK, Peiris D, et al Task shifting for non-communicable disease management in low and middle income countries—a systematic review. PLoS ONE. 2014;9(8):e103754 10.1371/journal.pone.0103754 25121789 PMC4133198

[12] He J, Irazola V, Mills KT, Poggio R, Beratarrechea A, Dolan J, et al Effect of a community health worker–led multicomponent intervention on blood pressure control in low-income patients in Argentina: a randomized clinical trial. JAMA. 2017;318(11):1016–25. 10.1001/jama.2017.11358 28975305 PMC5761321

[13] Neupane D, McLachlan CS, Mishra SR, Olsen MH, Perry HB, Karki A, et al Effectiveness of a lifestyle intervention led by female community health volunteers versus usual care in blood pressure reduction (COBIN): an open-label, cluster-randomised trial. Lancet Glob Health. 2018;6(1):e66–73. 10.1016/S2214-109X(17)30411-4 29241617

[14] Jafar TH, Silva A, Naheed A, Jehan I, Liang F, Assam PN, et al Control of blood pressure and risk attenuation: a public health intervention in rural Bangladesh, Pakistan, and Sri Lanka: feasibility trial results. J Hypertens. 2016;34(9):1872–81. 10.1097/HJH.0000000000001014 27488552

[15] Kar SS, Thakur JS, Jain S, Kumar R. Cardiovascular disease risk management in a primary health care setting of north India. Indian Heart J. 2008;60(1):19–25. 19212017

[16] Newman PM, Franke MF, Arrieta J, Carrasco H, Elliott P, Flores H, et al Community health workers improve disease control and medication adherence among patients with diabetes and/or hypertension in Chiapas, Mexico: an observational stepped-wedge study. BMJ Glob Health. 2018;3(1):e000566 10.1136/bmjgh-2017-000566 29527344 PMC5841495

[17] Jafar TH, Hatcher J, Poulter N, Islam M, Hashmi S, Qadri Z, et al Community-based interventions to promote blood pressure control in a developing country: a cluster randomized trial. Ann Intern Med. 2009;151(9):593–601. 10.7326/0003-4819-151-9-200911030-00004 19884620

[18] Peiris D, Praveen D, Mogulluru K, Ameer MA, Raghu A, Li Q, et al SMARThealth India: a stepped-wedge, cluster randomised controlled trial of a community health worker managed mobile health intervention for people assessed at high cardiovascular disease risk in rural India. PLoS ONE. 2019;14(3):e0213708 10.1371/journal.pone.0213708 30913216 PMC6435227

[19] Mendis S, Johnston SC, Fan W, Oladapo O, Cameron A, Faramawi MF. Cardiovascular risk management and its impact on hypertension control in primary care in low-resource settings: a cluster-randomized trial. Bull World Health Organ. 2010;88(6):412–9. 10.2471/BLT.08.062364 20539854 PMC2878142

[20] Riley SB, Marshall ES. Group visits in diabetes care: a systematic review. Diabetes Educ. 2010;36(6):936–44. 10.1177/0145721710385013 20974905

[21] Riddell MA, Joshi R, Oldenburg B, Chow C, Thankappan KR, Mahal A, et al Cluster randomised feasibility trial to improve the Control of Hypertension In Rural India (CHIRI): a study protocol. BMJ Open. 2016;6(10):e012404 10.1136/bmjopen-2016-012404 27855099 PMC5073516

[22] Government of Kerala State Planning Board. Human development report 2005. Thiruvananthapuram: Government of Kerala State Planning Board; 2005 [cited 2018 Nov 9]. Available from: http://planningcommission.nic.in/plans/stateplan/sdr_pdf/shdr_kerala05.pdf.

[23] Government of India. District census 2011. Government of India; 2015 [cited 2018 Nov 9]. Available from: http://www.census2011.co.in/district.php.

[24] Thrift AG, Evans RG, Kalyanram K, Kartik K, Fitzgerald SM, Srikanth V. Gender-specific effects of caste and salt on hypertension in poverty: a population-based study. J Hypertens. 2011;29(3):443–50. 10.1097/HJH.0b013e328341888c 21119531

[25] World Health Organization. WHO STEPS: surveillance manual: the WHO STEPwise approach to noncommunicable disease risk factor surveillance. Geneva: World Health Organization; 2017 [cited 2018 Oct 10]. Available from: http://www.who.int/ncds/surveillance/steps/STEPS_Manual.pdf.

[26] Abdel-All M, Thrift AG, Riddell M, Thankappan KRT, Mini GK, Chow CK, et al Evaluation of a training program of hypertension for accredited social health activists (ASHA) in rural India. BMC Health Serv Res. 2018;18(1):320 10.1186/s12913-018-3140-8 29720161 PMC5932780

[27] Råstam L, Berglund G, Isacsson SO, Rydén L. The Skaraborg hypertension project. III. Influence on blood pressure of a medical care program for hypertension. Acta Med Scand. 1986;219(3):261–9. 3486551

[28] Parker DR, Evangelou E, Eaton CB. Intraclass correlation coefficients for cluster randomized trials in primary care: the cholesterol education and research trial (CEART). Contemp Clin Trials. 2005;26(2):260–7. 10.1016/j.cct.2005.01.002 15837446

[29] Blood Pressure Lowering Treatment Trialists’ Collaboration. Effects of different blood-pressure-lowering regimens on major cardiovascular events: results of prospectively-designed overviews of randomised trials. Lancet. 2003;362(9395):1527–35. 10.1016/s0140-6736(03)14739-3 14615107

[30] White ND, Lenz TL, Smith K. Tool guide for lifestyle behavior change in a cardiovascular risk reduction program. Psychol Res Behav Manag. 2013;6:55–63. 10.2147/PRBM.S40490 23983496 PMC3751462

[31] Matsumura K, Arima H, Tominaga M, Ohtsubo T, Sasaguri T, Fujii K, et al Impact of antihypertensive medication adherence on blood pressure control in hypertension: the COMFORT study. QJM. 2013;106(10):909–14. 10.1093/qjmed/hct121 23696676

[32] Aucott L, Rothnie H, McIntyre L, Thapa M, Waweru C, Gray D. Long-term weight loss from lifestyle intervention benefits blood pressure?: a systematic review. Hypertension. 2009;54(4):756–62. 10.1161/HYPERTENSIONAHA.109.135178 19704106

[33] Giraudeau B, Ravaud P. Preventing bias in cluster randomised trials. PLoS Med. 2009;6(5):e1000065 10.1371/journal.pmed.1000065 19536323 PMC2668175

