# Supplementary figures and images for: Effectiveness of a scalable group-based education and monitoring program, delivered by health workers, to improve control of hypertension in rural India: A cluster randomised controlled trial
Source: PLoS Med. 2020 Jan 2;17(1):e1002997. doi: 10.1371/journal.pmed.1002997 (PMC6939905; doi:10.1371/journal.pmed.1002997)

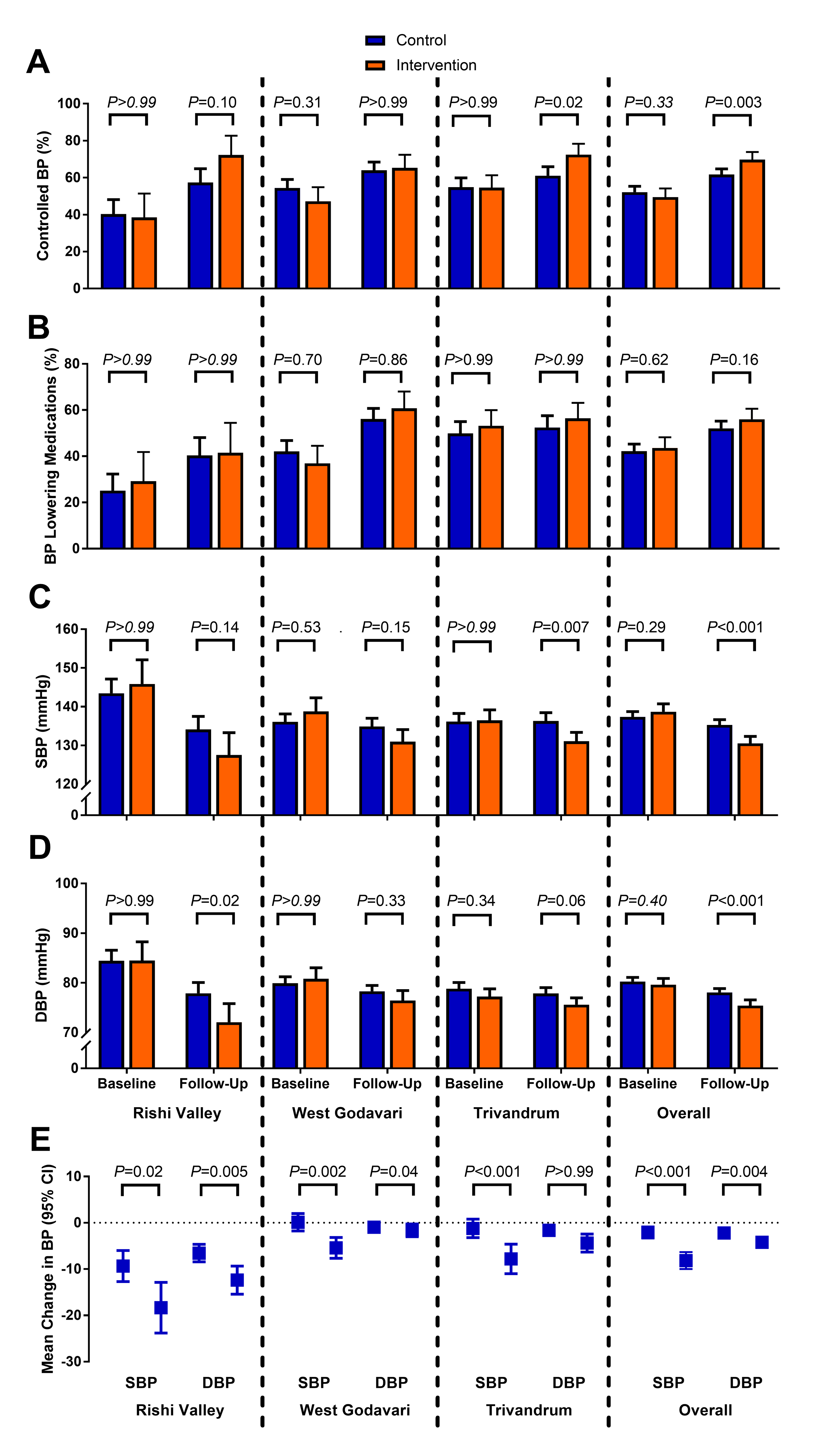

Supplement: S1 Fig — (A) Percent with controlled BP, (B) percent with BP-lowering medications, (C) mean SBP, (D) mean DBP, and (E) change in BP (mean change in mm Hg, and 95% confidence intervals). P values for categorical variables were generated using chi-squared tests (A and B) or linear regression for continuous variables (C–E), with Bonferroni correction for specific contrasts between each of the 3 regions. Error bars show 95% confidence limits. (TIF) [file pmed.1002997.s002.tif]

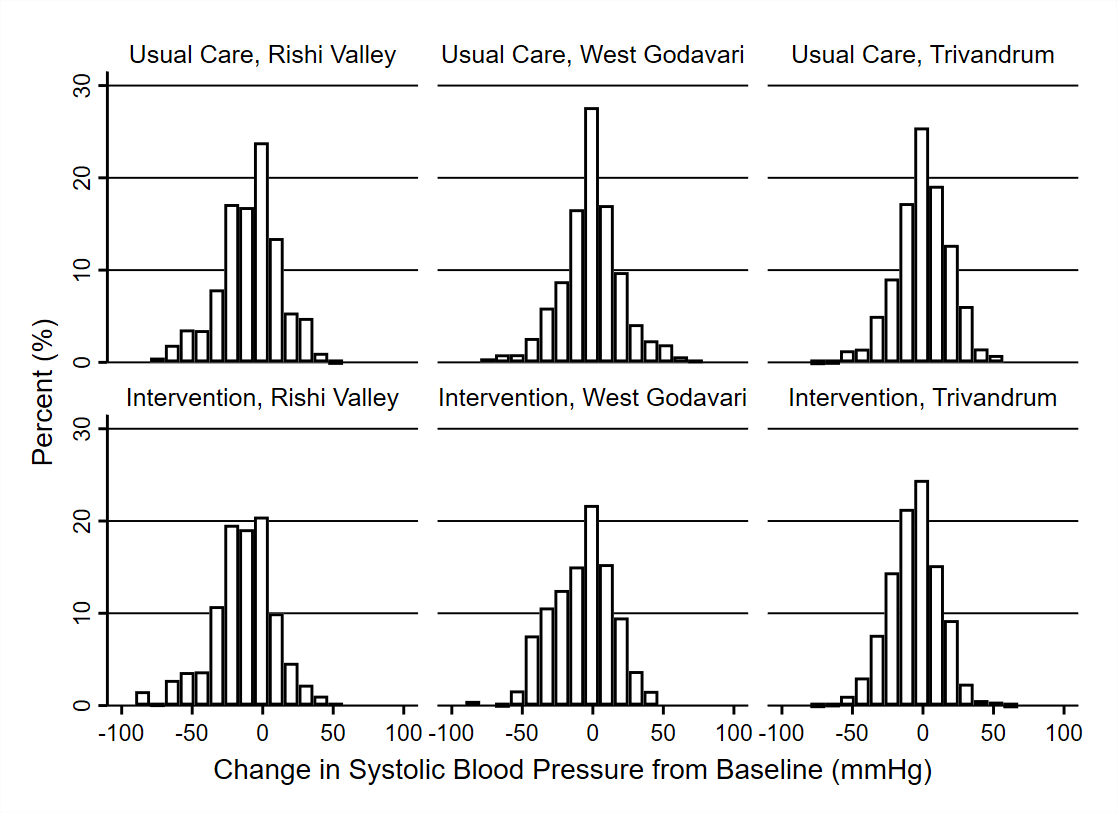

Supplement: S2 Fig — (TIF) [file pmed.1002997.s003.tif]

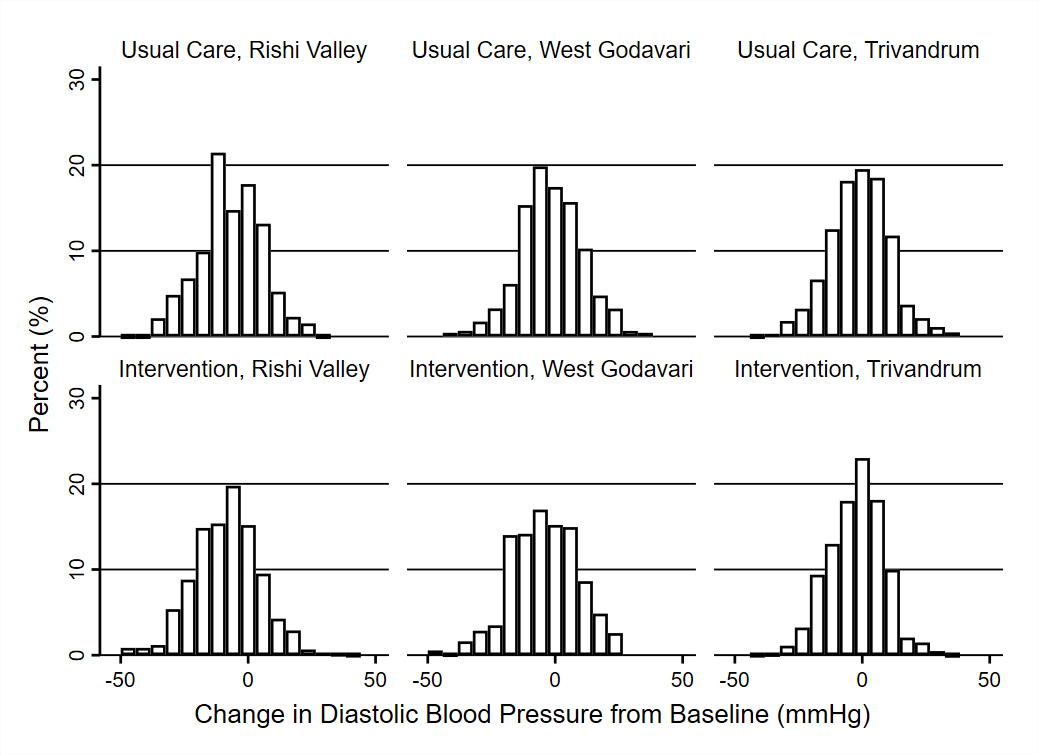

Supplement: S3 Fig — (TIF) [file pmed.1002997.s004.tif]

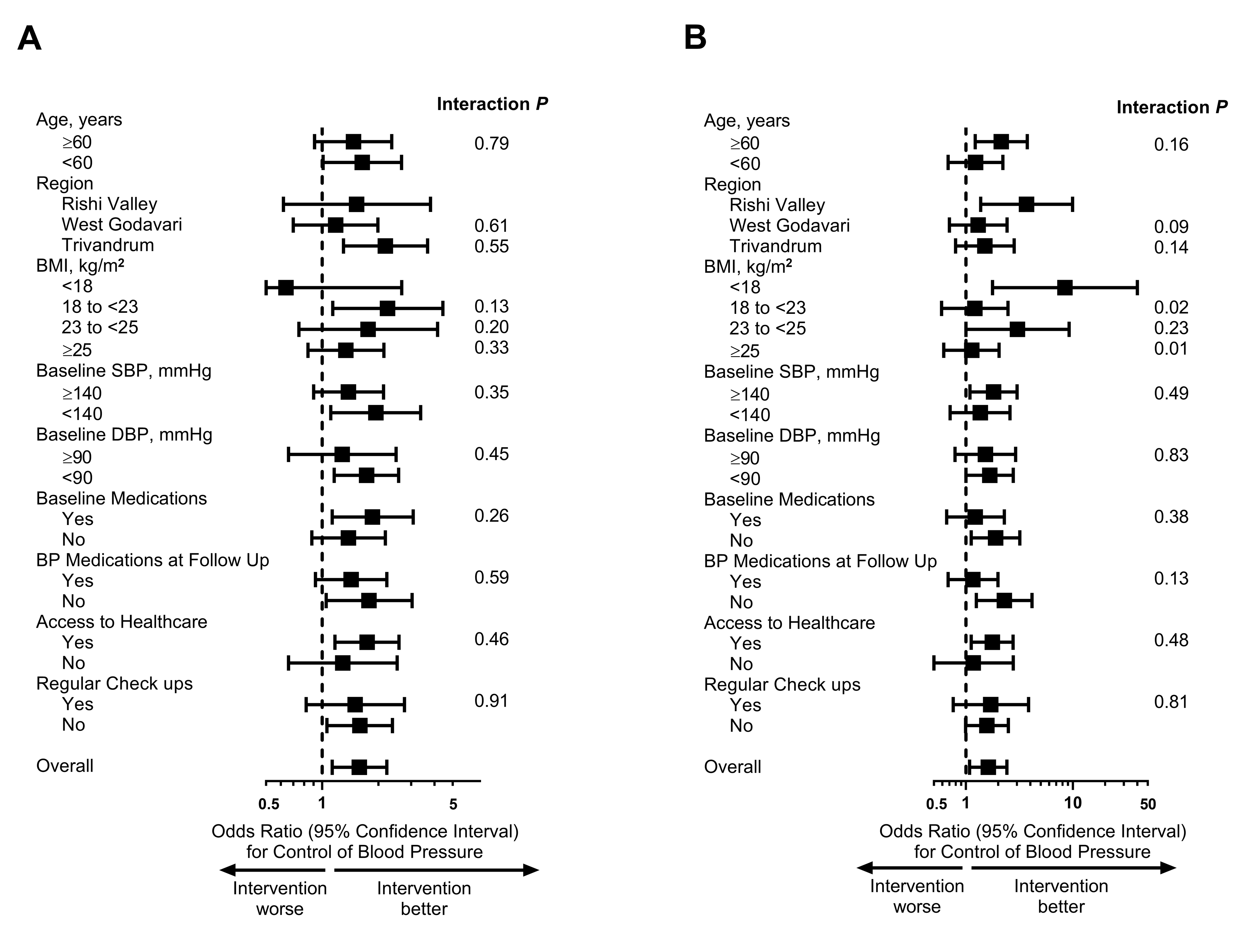

Supplement: S4 Fig — (A) Women; (B) men. The dashed line represents the line of no effect. Symbols show point estimates, and error bars show 95% confidence limits. P values indicate subgroup interactions (obtained using logistic regression). (TIF) [file pmed.1002997.s005.tif]
