# Supplementary material for: Effectiveness of a scalable group-based education and monitoring program, delivered by health workers, to improve control of hypertension in rural India: A cluster randomised controlled trial
Source: PLoS Med. 2020 Jan 2;17(1):e1002997. doi: 10.1371/journal.pmed.1002997 (PMC6939905; doi:10.1371/journal.pmed.1002997)
Supplement: S1 Table — (DOCX) [file pmed.1002997.s006.docx]

**S1 Table. Socioeconomic characteristics of participants in the intervention and UC groups in the three regions**

| **Variable** | **Rishi Valley** | |  | **West Godavari** | |  | **Trivandrum** | | ***P*_Region_** | ***P*_Treatment_** | ***P*_Region* Treatment_** |
| --- | --- | --- | --- | --- | --- | --- | --- | --- | --- | --- | --- |
|  | **Intervention**  **n = 135** | **UC**  **n = 213** |  | **Intervention**  **n =198** | **UC**  **n = 460** |  | **Intervention**  **n = 304** | **UC**  **n = 424** |  |  |  |
| Ability to read and write | 33 (25.0)‡ | 65 (30.7)* |  | 84 (42.4) | 221 (48.1)* |  | 266 (87.5)^E^ | 339 (80.0) | <0.001^D^ | 0.94 |  |
| Highest level of schooling | ¶ | § |  | * | § |  |  |  |  |  |  |
| No formal education | 72 (59.5) | 118 (56.5) |  | 75 (38.0) | 163 (35.8) |  | 40 (13.2)^G^ | 83 (19.6) | <0.001^D^ | 0.001 |  |
| Class 1 to 6 | 32 (26.5) | 52 (24.9) |  | 78 (39.6) | 185 (40.6) |  | 47 (15.5) | 107 (25.2) |  |  |  |
| Class 7 to 11 | 13 (10.7) | 33 (15.8) |  | 30 (15.2) | 73 (16.0) |  | 164 (54.0) | 176 (41.5) |  |  |  |
| Class 12+ | 4 (3.3) | 6 (2.9) |  | 14 (7.1) | 35 (7.7) |  | 53 (17.4) | 58 (13.7) |  |  |  |
| Above poverty line or no ration card | 7 (5.2)§ | 8 (3.8)§ |  | 25 (12.6) | 64 (13.9) |  | 193 (63.5) | 256 (60.4) | <0.001^D^ | 0.02 |  |
| People in household |  |  |  |  |  |  |  |  |  |  |  |
| Mean (SD) | 3.7 (2.5)§ | 4.2 (2.6)§ |  | 3.4 (1.8) | 3.7 (2.0) |  | 4.2 (1.7) | 3.9 (1.7) | <0.001^C^ | 0.10 | 0.002 |
| ≥ 5 people | 35 (26.7)§^E^ | 88 (42.1)§ |  | 48 (24.4) | 138 (30.0) |  | 125 (41.1) | 148 (34.9) | 0.001^AC^ | 0.57 |  |
| Visits to doctor | § | ‡ |  |  |  |  |  |  | <0.001^D^ | 0.33 |  |
| Never | 58 (44.3) | 97 (46.2) |  | 32 (16.2) | 57 (12.4) |  | 43 (14.1) | 53 (12.5) |  |  |  |
| Regular visits to doctor | 15 (11.5) | 28 (1332) |  | 26 (13.1) | 96 (20.9) |  | 108 (35.5) | 173 (40.8) |  |  |  |
| Irregular, but visited within past year | 31 (23.7) | 56 (26.7) |  | 102 (51.5) | 232 (50.4) |  | 125 (41.1) | 156 (36.8) |  |  |  |
| Not visited in past 1 year | 27 (20.6) | 29 (13.8) |  | 38 (19.2) | 75 (16.3) |  | 28 (9.2) | 42 (9.9) |  |  |  |
| Self-reported difficulty in accessing health care | 73 (56.2)‖ | 107 (51.0)‡ |  | 60 (30.3) | 120 (26.1)* |  | 16 (5.3)^G^ | 80 (18.9) | <0.001^D^ | 0.04 |  |

Data are presented as number (%) unless otherwise stated. UC, usual care; SD, standard deviation.

* 1 missing observation; †2 missing observations; ‡ 3 missing observations; §4 missing observations. ‖ 5 missing observations; ¶ 14 missing observations

*P*_Region_, *P*_Treatment_, and *P*_Region*Treatment_ were determined using ANOVA for continuous variables and χ^2^ test for categorical variables. For continuous variables, if *P*_Region_ ≤0.05, Tukey’s test was used to determine which regions differed at *P*≤0.05. For categorical variables, χ^2^ test was used with a Bonferroni correction for multiple comparisons (3 regions). This is shown by superscript (A = RV vs. WG, B = RV vs T, C = WG vs T, D = all differ).

If *P*_Treatment_ or *P*_Region*Treatment_ ≤0.05, intervention groups that differ significantly from their UC group are marked as followed (E p≤0.05, F *P*≤0.01, G *P*≤0.001), derived using Student’s unpaired t‐test or χ^2^ test, with Bonferroni correction for specific contrasts in each of the three regions.

Class 12+ includes individuals who graduated from secondary schooling, completed technical college or completed university.

Income level above the poverty line was assessed using self-reported data for use of a government issued ration card.
