# Supplementary material for: Effectiveness of a scalable group-based education and monitoring program, delivered by health workers, to improve control of hypertension in rural India: A cluster randomised controlled trial
Source: PLoS Med. 2020 Jan 2;17(1):e1002997. doi: 10.1371/journal.pmed.1002997 (PMC6939905; doi:10.1371/journal.pmed.1002997)
Supplement: S2 Table — (DOCX) [file pmed.1002997.s007.docx]

**S2 Table. Baseline characteristics of participants with hypertension in the intervention and usual care groups.**

| **Variable** | **Rishi Valley** | |  | **West Godavari** | |  | **Trivandrum** | | ***P*_Region_** | ***P*_Treatment_** | ***P*_Region* Treatment_** |
| --- | --- | --- | --- | --- | --- | --- | --- | --- | --- | --- | --- |
|  | **Intervention**  **n = 135** | **UC**  **n = 213** |  | **Intervention**  **n =198** | **UC**  **n = 460** |  | **Intervention**  **n = 304** | **UC**  **n = 424** |  |  |  |
| Age (years), mean (SD) | 58.7 (12.9) | 57.3 (12.9) |  | 56.2 (13.9) | 55.8 (13.8) |  | 55.8 (15.1) | 58.1 (14.3) | 0.11 | 0.07 | 0.20 |
| Females, n (%) | 76 (56.3) | 124 (58.2) |  | 119 (60.4)* | 258 (56.6)§ |  | 178 (58.6) | 251 (59.2) | 0.86 | 0.77 |  |
| SBP (mmHg), mean (SD) | 148.0 (26.8) | 144.8 (22.9) |  | 139.5 (22.9) | 136.2 (22.1) |  | 137.9 (19.7) | 136.0 (21.2) | <0.001^AB^ | 0.02 | 0.82 |
| DBP (mmHg), mean (SD) | 83.8 (14.4) | 85.9 (14.1) |  | 81.3 (14.8) | 79.9 (14.3) |  | 78.4 (12.1) | 78.6 (12.6) | <0.001^D^ | 0.66 | 0.19 |
| Controlled hypertension, n (%) | 47 (34.8) | 69 (32.4) |  | 88 (44.4) | 249 (54.1) |  | 142 (46.4) | 231 (54.5) | <0.001^AB^ | 0.01 |  |
| Antihypertensive medications, n (%) | 31 (23.0) | 43 (20.2) |  | 71 (35.9) | 194 (42.2) |  | 140 (46.1) | 208 (49.1) | <0.001^D^ | 0.29 |  |
| Body Mass Index (kg/m^2^), mean (SD) | 20.9 (3.8)*^F^ | 22.7 (4.9)‖ |  | 25.6 (4.6)* | 25.5 (4.8) |  | 25.5 (4.6) | 25.1 (4.9)* | <0.001^AB^ | 0.08 | 0.003 |
| Waist Hip Ratio, mean (SD) | 0.85 (0.09)¶ | 0.88 (0.10)§ |  | 0.92 (0.09)* | 0.92 (0.09) |  | 0.94 (0.09) | 0.95 (0.07) | <0.001^D^ | 0.07 | 0.09 |
| Physical activity per day (METS), mean (SD) | 1071 (1154)§^F^ | 743 (1087)§ |  | 955 (937) | 1030 (864) |  | 848 (844) | 734 (793) | <0.001^AC^ | 0.01 | 0.01 |
| Fruit (weekly serves), mean (SD) | 1.4 (3.0)§ | 1.6 (2.6)§ |  | 2.8 (4.0) | 2.3 (3.3)* |  | 4.5 (7.2)^F^ | 3.2 (3.5) | <0.001^D^ | 0.02 | 0.03 |
| Vegetables (weekly serves), mean (SD) | 15.8 (10.2)§ | 15.1 (8.5)§ |  | 8.3 (3.6) | 8.4 (3.5)* |  | ^–^ | ^–^ | <0.001^A^ | 0.40 | 0.32 |
| Teaspoons of salt added/day, mean (SD) | 0.05 (0.11)# | 0.04 (0.05)** |  | 0.1 (0.08) | 0.1 (0.2) |  | 0.01 (0.06) | 0.004 (0.03) | <0.001 | 0.31 | 0.54 |
| Adding extra salt to food, n (%) | 65 (50.8)# | 89 (49.7)** |  | 154 (77.8)^E^ | 311 (67.6) |  | 8 (2.6) | 10 (2.4) | <0.001^D^ | 0.30 |  |
| Current smoking, n (%) | 21 (16.0)§ | 31 (14.8)§ |  | 46 (23.5)† | 80 (17.5)† |  | 29 (9.6)† | 54 (12.7) | <0.001^C^ | 0.94 |  |
| Current alcohol use, n (%) | 7 (5.3)§ | 26 (12.4)§ |  | 28 (14.4)‡ | 56 (12.3)‡ |  | 33 (10.9)* | 63 (14.9) | 0.24 | 0.13 |  |

UC, Usual Care; SBP, systolic blood pressure; DBP, diastolic blood pressure; SD, standard deviation; METS, metabolic equivalent tasks.

* 1 missing observation; †2 missing observations; ‡ 3 missing observations; §4 missing observations; ‖ 5 missing observations; ¶ 6 missing observations; # 7 missing observations; ** 34 missing observations. The data for serves of vegetables in Trivandrum had some errors that could not be resolved. Therefore, there are no data for weekly serves of vegetables in this region.

Control of hypertension is defined as SBP < 140 mmHg and DBP < 90 mmHg; control may be achieved with use of antihypertensive agents or changing lifestyle.

*P*_Region_, *P*_Treatment_, and *P*_Region*Treatment_ were determined using ANOVA for continuous variables and χ^2^ test for categorical variables. For continuous variables, if *P*_Region_ ≤0.05, Tukey’s test was used to determine which regions differed at *P*≤0.05. For categorical variables, χ^2^ test was used with a Bonferroni correction for multiple comparisons (3 regions). This is shown by superscript (A = RV vs. WG, B = RV vs T, C = WG vs T, D = all differ).

If *P*_Treatment_ or *P*_Region*Treatment_ ≤0.05, intervention groups that differ significantly from their UC group are marked as followed (E *P*≤0.05, F *P<*0.01, G *P<*0.001), derived using Student’s unpaired t‐test or χ^2^ test, with Bonferroni correction for specific contrasts in each of the three regions.
