# Supplementary material for: Effectiveness of a scalable group-based education and monitoring program, delivered by health workers, to improve control of hypertension in rural India: A cluster randomised controlled trial
Source: PLoS Med. 2020 Jan 2;17(1):e1002997. doi: 10.1371/journal.pmed.1002997 (PMC6939905; doi:10.1371/journal.pmed.1002997)
Supplement: S3 Table — (DOCX) [file pmed.1002997.s008.docx]

**S3 Table. Effects of the intervention on control of hypertension (primary outcome) and prescription of antihypertensive medications (secondary outcome) in people with hypertension: Imputation analysis using intention-to-treat principles**

| **Variables** | **Number of Participants** | |  | **Change from baseline to follow-up*** | | ***P*** |  | **Odds Ratio (95% confidence interval)†** | ***P*** |
| --- | --- | --- | --- | --- | --- | --- | --- | --- | --- |
|  | **Intervention** | **UC‡** |  | **Intervention** | **UC** |  |  |  |  |
| **Overall** |  |  |  |  |  |  |  |  |  |
| Control of hypertension | 637 | 1,097 |  | 142 (22.3) | 124 (11.3) | <0.001 |  | 1.4 (1.1 – 1.8) | 0.007 |
| Prescribed antihypertensive medications | 637 | 1,097 |  | 88 (13.8) | 116 (10.6) | 0.04 |  | 1.2 (0.8 – 1.7) | 0.38 |
| **Women** |  |  |  |  |  |  |  |  |  |
| Control of hypertension | 373 | 633 |  | 74 (19.8) | 63 (9.9) | <0.001 |  | 1.4 (1.0 – 2.0) | 0.05 |
| Prescribed antihypertensive medications | 373 | 633 |  | 53 (14.2) | 51 (8.1) | 0.002 |  | 1.4 (0.9 – 2.1) | 0.11 |
| **Men** |  |  |  |  |  |  |  |  |  |
| Control of hypertension | 263 | 460 |  | 68 (25.9) | 62 (13.5) | <0.001 |  | 1.4 (1.0 – 2.1) | 0.06 |
| Prescribed antihypertensive medications | 263 | 460 |  | 35 (13.3) | 65 (14.1) | 0.76 |  | 0.9 (0.5 – 1.7) | 0.85 |

UC, Usual Care. There are 4 missing observations for sex in usual care and one in the intervention group.

* Change in control of hypertension was obtained by subtracting the number of people with control of hypertension at baseline from the number with control at follow-up. The same approach was applied to prescription of antihypertensive medications. Positive number demonstrates improvement.

† Odds Ratios obtained using mixed-effects logistic regression, clustered by village and study region. For control of hypertension, the dependent variable was control of hypertension at follow-up, with adjustment for control of hypertension at baseline (ICC: overall <0.001; women <0.001; men <0.001). The same approach was applied to prescription of antihypertensive medications (ICC: overall 0.02; women 0.004; men 0.04).

Controlled hypertension at baseline and mean systolic blood pressure at baseline were used to impute data for controlled hypertension at follow-up (Women: 37 UC, 93 intervention; Men: 49 UC, 85 Intervention); blood pressure medications at baseline were used to impute data for blood pressure medications at follow-up (Women: 36 UC, 93 intervention; Men: 49 UC, 85 Intervention)
