# Supplementary material for: Effectiveness of a scalable group-based education and monitoring program, delivered by health workers, to improve control of hypertension in rural India: A cluster randomised controlled trial
Source: PLoS Med. 2020 Jan 2;17(1):e1002997. doi: 10.1371/journal.pmed.1002997 (PMC6939905; doi:10.1371/journal.pmed.1002997)
Supplement: S4 Table — (DOCX) [file pmed.1002997.s009.docx]

**S4 Table. Sensitivity analysis of the effects of intervention on the primary outcome (control of hypertension) in people with hypertension, excluding those with controlled hypertension at baseline**

| **Control of Hypertension** | **Number of Participants** | |  | **Controlled hypertension at follow-up** | | ***P*** |  | **Odds Ratio (95% confidence interval)*** | ***P*** |
| --- | --- | --- | --- | --- | --- | --- | --- | --- | --- |
|  | **Intervention** | **UC†** |  | **Intervention** | **UC** |  |  |  |  |
| **Overall** | 360 | 548 |  | 178 (49.5) | 232 (42.3) | 0.04 |  | 1.3 (1.0 – 1.8) | 0.06 |
|  |  |  |  |  |  |  |  |  |  |
| **Women** | 195 | 295 |  | 93 (47.5) | 130 (44.0) | 0.43 |  | 1.2 (0.8 – 1.7) | 0.49 |
|  |  |  |  |  |  |  |  |  |  |
| **Men** | 165 | 252 |  | 85 (51.8) | 102 (40.4) | 0.03 |  | 1.6 (1.0 – 2.5) | 0.04 |

UC, Usual Care.

* Odds Ratios obtained using mixed-effects logistic regression, clustered by village and study region. The dependent variable was control of hypertension at follow-up. When there were missing observations for control of blood pressure at follow-up, they were imputed from control of blood pressure at baseline and mean systolic blood pressure at baseline (Women: 29 in the usual care group, 62 in the intervention group; Men: 35 in the usual care group, 66 in the intervention group). The results of the regression for men show the odds ratio and 95% confidence limits for an analysis clustered by village alone. The identical results occurred when clustered by region alone (the analysis would not iterate for the combined clustering of village and region).
