# Supplementary material for: Effectiveness of a scalable group-based education and monitoring program, delivered by health workers, to improve control of hypertension in rural India: A cluster randomised controlled trial
Source: PLoS Med. 2020 Jan 2;17(1):e1002997. doi: 10.1371/journal.pmed.1002997 (PMC6939905; doi:10.1371/journal.pmed.1002997)
Supplement: S5 Table — (DOCX) [file pmed.1002997.s010.docx]

**S5 Table. Changes in risk factors from baseline to follow-up in the intervention and usual care groups (categorical variables)**

|  | **Rishi Valley**  **n (%)** | |  | **West Godavari**  **n (%)** | |  | **Trivandrum**  **n (%)** | |
| --- | --- | --- | --- | --- | --- | --- | --- | --- |
| **Variables** | **Intervention**  **n = 135** | **UC**  **n = 213** |  | **Intervention**  **n =198** | **UC**  **n = 460** |  | **Intervention**  **n = 304** | **UC**  **n = 424** |
| Change in control of hypertension^2^ | 36 (27.0) | 50 (23.5) |  | 40 (20.1)^G^ | 45 (9.8) |  | 66 (21.7)^G^ | 30 (7.0) |
| Change in prescribed antihypertensive medications^2^ | 22 (16.4) | 38 (17.7) |  | 45 (22.8)^F^ | 64 (13.9) |  | 21 (6.9) | 15 (3.4) |
| Change in adding extra salt to food^1^ | -32 (-25.2)‖ | -53 (-29.5)¶ |  | -41 (-20.8)^G^ | -22 (-4.7) |  | 4 (1.3) | 1 (0.3) |
| Change in current smoking^1^ | -5 (-3.8)§ | -1 (-0.5)§ |  | -7 (-3.8)†^G^ | 2 (0.4)† |  | -6 (-2.0)† | -7 (-1.7) |
| Change alcohol use in last 30 days^1^ | -1 (-1.0)§ | -6 (-3.0)§ |  | -3 (-1.7)‡^E^ | 13 (2.8)‡ |  | -2 (-0.7)* | -4 (-1.0) |

UC, Usual Care.

^1^ Negative number demonstrates improvement, ^2^ Positive number demonstrates improvement

* 1 missing observation; †2 missing observations; ‡ 3 missing observations; §4 missing observations. ‖ 7 missing observations; ¶ 34 missing observations.

Change in control of hypertension was obtained by subtracting the number of people with control of hypertension at baseline from the number with control at follow-up. This same approach was applied for all other variables in the table.

Intervention groups that differ significantly from their UC group are marked as follows (E p≤0.05, F *P<*0.01, G *P<*0.001), derived using χ^2^ test, with Bonferroni correction for specific contrasts in each of the three regions.

Controlled hypertension at baseline and mean systolic blood pressure at baseline were used to impute data for controlled hypertension at follow-up (Women: 37 UC, 93 intervention; Men: 49 UC, 85 Intervention); blood pressure medications at baseline were used to impute data for blood pressure medications at follow-up (Women: 36 UC, 93 intervention; Men: 49 UC, 85 Intervention). Data for salt at follow-up were imputed for 254 observations (using salt at baseline); Data for smoking at follow-up were imputed for 258 observations (using smoking at baseline and sex); Data for consuming alcohol at follow-up were imputed for 258 observations (using alcohol consumption at baseline and sex).

The number of people in these analyses are rounded to the nearest whole number as, with imputation analysis, the number of people are an average of 20 imputation databases and so are not usually in whole numbers. This means that sometimes the percentages do not always exactly reflect the whole numbers provided.
