# Supplementary material for: Effectiveness of a scalable group-based education and monitoring program, delivered by health workers, to improve control of hypertension in rural India: A cluster randomised controlled trial
Source: PLoS Med. 2020 Jan 2;17(1):e1002997. doi: 10.1371/journal.pmed.1002997 (PMC6939905; doi:10.1371/journal.pmed.1002997)
Supplement: S6 Table — (DOCX) [file pmed.1002997.s011.docx]

**S6 Table. Changes in control of blood pressure and use of antihypertensive medications from baseline to follow-up in women and men in the intervention and usual care groups.**

| **Variables, n (%)** | **Rishi Valley** | |  | **West Godavari** | |  | **Trivandrum** | |
| --- | --- | --- | --- | --- | --- | --- | --- | --- |
|  | **Intervention** | **UC** |  | **Intervention*** | **UC**† |  | **Intervention** | **UC** |
| Women | **n = 76** | **n = 124** |  | **n =119** | **n = 258** |  | **n = 178** | **n = 251** |
| Change in control of hypertension (%)^1^ | 18 (23.9) | 32 (25.9) |  | 18 (15.1)^F^ | 15 (5.8) |  | 38 (21.5)^G^ | 16 (6.4) |
| Change in prescribed antihypertensive medications (%)^1^ | 9 (12.0) | 23 (18.7) |  | 29 (24.5)^G^ | 20 (7.7) |  | 15 (8.4)^E^ | 7 (3.0) |
|  |  |  |  |  |  |  |  |  |
| Men | **n = 59** | **n = 89** |  | **n = 78** | **n = 198** |  | **n = 126** | **n = 173** |
| Change in control of hypertension (%)^1^ | 18 (31.0) | 18 (20.1) |  | 22 (27.9)^E^ | 31 (15.7) |  | 28 (21.9)^F^ | 14 (7.8) |
| Change in prescribed antihypertensive medications (%)^1^ | 13 (22.0) | 15 (16.3) |  | 16 (20.4) | 43 (21.7) |  | 6 (4.7) | 7 (4.1) |

UC, Usual Care.

^1^Positive number denotes improvement. Change in control of hypertension was obtained by subtracting the number of people with control of hypertension at baseline from the number with control at follow-up. This same approach was applied for change in prescribed antihypertensive medications. Controlled hypertension at baseline and mean systolic blood pressure at baseline were used to impute data for controlled hypertension at follow-up (Women: 36 UC, 93 intervention; Men: 49 UC, 85 Intervention); blood pressure medications at baseline were used to impute data for blood pressure medications at follow-up (Women: 36 UC, 93 intervention; Men: 49 UC, 85 Intervention).

* 1 missing observation for sex; † 4 missing observations for sex

Intervention groups that differ significantly from their UC group are marked as follows (E *P*≤0.05, F *P*≤0.01, G *P*≤0.001), derived using test for differences in proportions, with Bonferroni correction for specific contrasts in each of the three regions.

The number of people in these analyses are rounded to the nearest whole number as, with imputation analysis, the number of people are an average of 20 imputation databases and so are not usually in whole numbers. This means that sometimes the percentages do not always exactly reflect the whole numbers provided.
