# Supplementary material for: Effectiveness of a scalable group-based education and monitoring program, delivered by health workers, to improve control of hypertension in rural India: A cluster randomised controlled trial
Source: PLoS Med. 2020 Jan 2;17(1):e1002997. doi: 10.1371/journal.pmed.1002997 (PMC6939905; doi:10.1371/journal.pmed.1002997)
Supplement: S7 Table — (DOCX) [file pmed.1002997.s012.docx]

**S7 Table. Effects of the intervention on secondary outcomes in people with hypertension: Imputation analysis using intention-to-treat principles**

| **Variables^1^** | **Number of Participants** | |  | **Unadjusted Mean Change (95% Confidence Interval)** | | **Unadjusted Net Mean Change** | ***P*** |
| --- | --- | --- | --- | --- | --- | --- | --- |
|  | **Intervention** | **UC** |  | **Intervention** | **UC** | **(95% Confidence Interval)** |  |
| **Overall** |  |  |  |  |  |  |  |
| SBP (mmHg) | 637 | 1,097 |  | -8.0 (-10.0 – -6.0) | -2.4 (-3.6 – -1.1) | -5.7 (-8.7 – -2.6) | <0.001 |
| DBP (mmHg) | 637 | 1,097 |  | -4.3 (-5.4 – -3.1) | -2.4 (-3.1 – -1.6) | -1.9 (-3.5 – -0.1) | 0.01 |
| **Women** |  |  |  |  |  |  |  |
| SBP (mmHg) | 373 | 633 |  | -6.7 (-9.1 – -4.4) | -1.8 (-3.4 – -0.1) | -4.9 (-8.1 – -1.7) | 0.003 |
| DBP (mmHg) | 373 | 633 |  | -2.7 (-4.1 – -1.4) | -1.8 (-2.8 – -0.9) | -0.9 (-2.5 – 0.6) | 0.25 |
| **Men** |  |  |  |  |  |  |  |
| SBP (mmHg) | 263 | 460 |  | -9.8 (-12.9 – -6.8) | -3.3 (-5.4 – -1.3) | -6.9 (-11.6 – -2.2) | 0.004 |
| DBP (mmHg) | 263 | 460 |  | -6.4 (-8.3 – -4.6) | -3.2 (-4.5 – -2.0) | -3.4 (-6.0 – -0.8) | 0.01 |

UC, usual care; SBP, systolic blood pressure; DBP, diastolic blood pressure. There are 4 missing observations for sex in usual care and one in the intervention group.

^1^ Negative number demonstrates improvement.

Mean systolic and diastolic blood pressure at baseline were used to impute change in systolic blood pressure from baseline to follow-up (269 observations), and change in diastolic blood pressure from baseline to follow-up (269 observations).

Analyses for unadjusted net mean change were conducted using mixed-effects linear regression, clustered by region and village. Intra-class correlation (ICC) for SBP is 0.008 overall, 0.001 for women, and 0.02 for men; ICC for DBP is 0.002 overall, <0.001 for women, and 0.01 for men.
