# Supplementary material for: Effectiveness of a scalable group-based education and monitoring program, delivered by health workers, to improve control of hypertension in rural India: A cluster randomised controlled trial
Source: PLoS Med. 2020 Jan 2;17(1):e1002997. doi: 10.1371/journal.pmed.1002997 (PMC6939905; doi:10.1371/journal.pmed.1002997)
Supplement: S8 Table — (DOCX) [file pmed.1002997.s013.docx]

**S8 Table. Changes in risk factors from baseline to follow-up in the intervention and usual care groups (continuous variables).**

| **Variables** | **Rishi Valley**  **mean (SD)** | |  | **West Godavari**  **mean (SD)** | |  | **Trivandrum**  **mean (SD)** | | **P_Region_** | **P_treatment_** | **P_Region* Treatment_** |
| --- | --- | --- | --- | --- | --- | --- | --- | --- | --- | --- | --- |
|  | **Intervention**  **n = 135** | **UC**  **n = 213** |  | **Intervention**  **n =198** | **UC**  **n = 460** |  | **Intervention**  **n = 304** | **UC**  **n = 424** |  |  |  |
| SBP (mmHg)^1^ | -13.6 (29.6) | -9.5 (22.8) |  | -7.8 (21.8)^F^ | -1.3 (21.8) |  | -5.6 (21.2)^G^ | 0.02 (19.4) | <0.001^AB^ | <0.001 | >0.50 |
| DBP (mmHg)^1^ | -8.5 (15.9) | -6.8 (13.3) |  | -4.5 (13.6)^E^ | -1.6 (12.4) |  | -2.2 (12.3) | -1.0 (12.3) | <0.001^AB^ | 0.28 | >0.50 |
| Body Mass Index (kg/m^2^)^1^ | 0.20 (1.9)* | 0.003 (1.72)‖ |  | 0.37 (1.7)* | 0.54 (1.9) |  | 0.03 (1.8) | 0.02 (1.7)* |  |  |  |
| Waist Hip Ratio^1^ | 0.014 (0.08)¶ | 0.003 (0.07)¶ |  | 0.014 (0.066)* | 0.015 (0.055) |  | -0.002 (0.069) | -0.011 (0.066) |  |  |  |
| Physical activity (METS) per day^2^ | 112 (214)§ | 103 (143)§ |  | 161 (181) | 152 (181) |  | 63 (164) | 64 (156) |  |  |  |
| Fruit (weekly serves)^2^ | 0.82 (4.52)§ | 0.55 (4.04)§ |  | 0.85 (5.19) | 0.85 (4.10)* |  | -0.85 (7.58) | 0.04 (3.88) |  |  |  |

Data are presented as mean (standard deviation). UC, Usual Care; SBP, systolic blood pressure; DBP, diastolic blood pressure; SD, standard deviation; METS, metabolic equivalent tasks.

^1^ Negative number demonstrates improvement, ^2^ Positive number demonstrates improvement.

* 1 missing observation; †2 missing observations; ‡ 3 missing observations; §4 missing observations. ‖ 5 missing observations; ¶ 6 missing observations

Mean systolic and diastolic blood pressure at baseline were used to impute the change in systolic and diastolic blood pressure from baseline to follow-up (269 observations), and diastolic blood pressure at follow-up (269 observations). Change in body mass index (BMI) between baseline and follow-up was imputed for 263 observations (using BMI at baseline); change in waist-hip ratio (WHR) was imputed for 263 observations (using WHR and BMI at baseline); change in physical activity per day was imputed for 263 observations (using physical activity at baseline); change in fruit consumption per week imputed for 263 observations (using fruit consumption at baseline)

*P*_Region_, *P*_Treatment_, and *P*_Region*Treatment_ were determined using linear regression. Regions which differed are shown by superscripts (A = Rishi Valley vs. West Godavari, B = Rishi Valley vs Trivandrum, C = West Godavari vs Trivandrum, D = all differ).

If *P*_Treatment_ or *P*_Region*Treatment_ ≤0.05, intervention groups that differ significantly from their UC group are marked as followed (E p≤0.05, F *P<*0.01, G *P<*0.001), derived using linear regression, with Bonferroni correction for specific contrasts in each of the three regions.

The number of people in these analyses are rounded to the nearest whole number as, with imputation analysis, the number of people are an average of 20 imputation databases and so are not usually in whole numbers. This means that sometimes the percentages do not always exactly reflect the whole numbers provided.
