# Supplementary material for: Effectiveness of a scalable group-based education and monitoring program, delivered by health workers, to improve control of hypertension in rural India: A cluster randomised controlled trial
Source: PLoS Med. 2020 Jan 2;17(1):e1002997. doi: 10.1371/journal.pmed.1002997 (PMC6939905; doi:10.1371/journal.pmed.1002997)
Supplement: S9 Table — (DOCX) [file pmed.1002997.s014.docx]

**S9 Table. Changes in blood pressure from baseline to follow-up in women and men in the intervention and usual care groups: imputation analysis using intention-to-treat principles.**

| **Variables, mean (SD)** | **Rishi Valley** | |  | **West Godavari*** | |  | **Trivandrum** | |
| --- | --- | --- | --- | --- | --- | --- | --- | --- |
|  | **Intervention** | **UC** |  | **Intervention** | **UC** |  | **Intervention** | **UC** |
| Women | **n = 76** | **n = 124** |  | **n =119** | **n = 258** |  | **n = 178** | **n = 251** |
| SBP (mmHg)^1^ | -10.0 (27.8) | -10.5 (21.8) |  | -5.4 (19.9) | 0.1 (21.5) |  | -6.3 (19.2)^G^ | 0.6 (18.6) |
| DBP (mmHg)^1^ | -6.3 (14.8) | -7.0 (12.7) |  | -2.5 (12.3) | -0.9 (11.0) |  | -1.4 (11.3) | -0.3 (11.7) |
|  |  |  |  |  |  |  |  |  |
| Men | **n = 59** | **n = 89** |  | **n =78** | **n = 198** |  | **n = 126** | **n = 173** |
| SBP (mmHg)^1^ | -18.4 (28.6) | -8.2 (25.4) |  | -11.6 (24.0)^E^ | -3.3 (22.1) |  | -4.7 (21.3) | -0.9 (21.5) |
| DBP (mmHg)^1^ | -11.3 (15.3) | -6.6 (14.2) |  | -7.7 (14.8)^E^ | -2.9 (14.0) |  | -2.0 (12.6) | -1.9 (13.5) |

UC, Usual Care; SBP, systolic blood pressure; DBP, diastolic blood pressure; SD, standard deviation.

* 5 missing observations for Sex in West Godavari.

^1^Negative number denotes improvement

Mean systolic and diastolic blood pressure at baseline were used to impute the change in systolic and diastolic blood pressure from baseline to follow-up (263 observations), and diastolic blood pressure at follow-up (263 observations).

Intervention groups that differ significantly from the corresponding usual care group are marked as follows (E *P*≤0.05, F *P*≤0.01, G *P*≤0.001), derived using regression, with Bonferroni correction for specific contrasts in each of the three regions.

The number of people in these analyses are rounded to the nearest whole number as, with imputation analysis, the number of people are an average of 20 imputation databases and so are not usually in whole numbers. This means that sometimes the percentages do not always exactly reflect the whole numbers provided.
